# Measurement of multijet azimuthal correlations and determination of the strong coupling in proton-proton collisions at $\sqrt{s}=13\,\text {Te}\hspace{-.08em}\text {V}$

**DOI:** 10.1140/epjc/s10052-024-13116-7

**Published:** 2024-08-21

**Authors:** CMS Collaboration, A. Hayrapetyan, A. Tumasyan, W. Adam, J. W. Andrejkovic, T. Bergauer, S. Chatterjee, K. Damanakis, M. Dragicevic, P. S. Hussain, M. Jeitler, N. Krammer, A. Li, D. Liko, I. Mikulec, J. Schieck, R. Schöfbeck, D. Schwarz, M. Sonawane, S. Templ, W. Waltenberger, C.-E. Wulz, M. R. Darwish, T. Janssen, P. Van Mechelen, E. S. Bols, J. D’Hondt, S. Dansana, A. De Moor, M. Delcourt, H. El Faham, S. Lowette, I. Makarenko, D. Müller, A. R. Sahasransu, S. Tavernier, M. Tytgat, G. P. Van Onsem, S. Van Putte, D. Vannerom, B. Clerbaux, A. K. Das, G. De Lentdecker, L. Favart, D. Hohov, J. Jaramillo, A. Khalilzadeh, K. Lee, M. Mahdavikhorrami, A. Malara, S. Paredes, L. Pétré, N. Postiau, L. Thomas, M. Vanden Bemden, C. Vander Velde, P. Vanlaer, M. De Coen, D. Dobur, Y. Hong, J. Knolle, L. Lambrecht, G. Mestdach, K. Mota Amarilo, C. Rendón, A. Samalan, K. Skovpen, N. Van Den Bossche, J. van der Linden, L. Wezenbeek, A. Benecke, A. Bethani, G. Bruno, C. Caputo, C. Delaere, I. S. Donertas, A. Giammanco, K. Jaffel, Sa. Jain, V. Lemaitre, J. Lidrych, P. Mastrapasqua, K. Mondal, T. T. Tran, S. Wertz, G. A. Alves, E. Coelho, C. Hensel, T. Menezes De Oliveira, A. Moraes, P. Rebello Teles, M. Soeiro, W. L. Aldá Júnior, M. Alves Gallo Pereira, M. Barroso Ferreira Filho, H. Brandao Malbouisson, W. Carvalho, J. Chinellato, E. M. Da Costa, G. G. Da Silveira, D. De Jesus Damiao, S. Fonseca De Souza, R. Gomes De Souza, J. Martins, C. Mora Herrera, L. Mundim, H. Nogima, A. Santoro, A. Sznajder, M. Thiel, A. Vilela Pereira, C. A. Bernardes, L. Calligaris, T. R. Fernandez Perez Tomei, E. M. Gregores, P. G. Mercadante, S. F. Novaes, B. Orzari, Sandra S. Padula, A. Aleksandrov, G. Antchev, R. Hadjiiska, P. Iaydjiev, M. Misheva, M. Shopova, G. Sultanov, A. Dimitrov, L. Litov, B. Pavlov, P. Petkov, A. Petrov, E. Shumka, S. Keshri, S. Thakur, T. Cheng, Q. Guo, T. Javaid, L. Yuan, Z. Hu, J. Liu, K. Yi, G. M. Chen, H. S. Chen, M. Chen, F. Iemmi, C. H. Jiang, A. Kapoor, H. Liao, Z.-A. Liu, R. Sharma, J. N. Song, J. Tao, C. Wang, J. Wang, Z. Wang, H. Zhang, A. Agapitos, Y. Ban, A. Levin, C. Li, Q. Li, Y. Mao, S. J. Qian, X. Sun, D. Wang, H. Yang, L. Zhang, C. Zhou, Z. You, N. Lu, G. Bauer, X. Gao, D. Leggat, H. Okawa, Z. Lin, C. Lu, M. Xiao, C. Avila, D. A. Barbosa Trujillo, A. Cabrera, C. Florez, J. Fraga, J. A. Reyes Vega, J. Mejia Guisao, F. Ramirez, M. Rodriguez, J. D. Ruiz Alvarez, D. Giljanovic, N. Godinovic, D. Lelas, A. Sculac, M. Kovac, T. Sculac, P. Bargassa, V. Brigljevic, B. K. Chitroda, D. Ferencek, S. Mishra, A. Starodumov, T. Susa, A. Attikis, K. Christoforou, S. Konstantinou, J. Mousa, C. Nicolaou, F. Ptochos, P. A. Razis, H. Rykaczewski, H. Saka, A. Stepennov, M. Finger, M. Finger, A. Kveton, E. Ayala, E. Carrera Jarrin, A. A. Abdelalim, E. Salama, M. A. Mahmoud, Y. Mohammed, K. Ehataht, M. Kadastik, T. Lange, S. Nandan, C. Nielsen, J. Pata, M. Raidal, L. Tani, C. Veelken, H. Kirschenmann, K. Osterberg, M. Voutilainen, S. Bharthuar, E. Brücken, F. Garcia, K. T. S. Kallonen, R. Kinnunen, T. Lampén, K. Lassila-Perini, S. Lehti, T. Lindén, L. Martikainen, M. Myllymäki, M. m. Rantanen, H. Siikonen, E. Tuominen, J. Tuominiemi, P. Luukka, H. Petrow, M. Besancon, F. Couderc, M. Dejardin, D. Denegri, J. L. Faure, F. Ferri, S. Ganjour, P. Gras, G. Hamel de Monchenault, V. Lohezic, J. Malcles, J. Rander, A. Rosowsky, M. Ö. Sahin, A. Savoy-Navarro, P. Simkina, M. Titov, M. Tornago, C. Baldenegro Barrera, F. Beaudette, A. Buchot Perraguin, P. Busson, A. Cappati, C. Charlot, F. Damas, O. Davignon, A. De Wit, B. A. Fontana Santos Alves, S. Ghosh, A. Gilbert, R. Granier de Cassagnac, A. Hakimi, B. Harikrishnan, L. Kalipoliti, G. Liu, J. Motta, M. Nguyen, C. Ochando, L. Portales, R. Salerno, J. B. Sauvan, Y. Sirois, A. Tarabini, E. Vernazza, A. Zabi, A. Zghiche, J.-L. Agram, J. Andrea, D. Apparu, D. Bloch, J.-M. Brom, E. C. Chabert, C. Collard, S. Falke, U. Goerlach, C. Grimault, R. Haeberle, A.-C. Le Bihan, M. Meena, G. Saha, M. A. Sessini, P. Van Hove, S. Beauceron, B. Blancon, G. Boudoul, N. Chanon, J. Choi, D. Contardo, P. Depasse, C. Dozen, H. El Mamouni, J. Fay, S. Gascon, M. Gouzevitch, C. Greenberg, G. Grenier, B. Ille, I. B. Laktineh, M. Lethuillier, L. Mirabito, S. Perries, A. Purohit, M. Vander Donckt, P. Verdier, J. Xiao, I. Lomidze, T. Toriashvili, Z. Tsamalaidze, V. Botta, L. Feld, K. Klein, M. Lipinski, D. Meuser, A. Pauls, N. Röwert, M. Teroerde, S. Diekmann, A. Dodonova, N. Eich, D. Eliseev, F. Engelke, J. Erdmann, M. Erdmann, P. Fackeldey, B. Fischer, T. Hebbeker, K. Hoepfner, F. Ivone, A. Jung, M. y. Lee, L. Mastrolorenzo, F. Mausolf, M. Merschmeyer, A. Meyer, S. Mukherjee, D. Noll, A. Novak, F. Nowotny, A. Pozdnyakov, Y. Rath, W. Redjeb, F. Rehm, H. Reithler, U. Sarkar, V. Sarkisovi, A. Schmidt, A. Sharma, J. L. Spah, A. Stein, F. Torres Da Silva De Araujo, L. Vigilante, S. Wiedenbeck, S. Zaleski, C. Dziwok, G. Flügge, W. Haj Ahmad, T. Kress, A. Nowack, O. Pooth, A. Stahl, T. Ziemons, A. Zotz, H. Aarup Petersen, M. Aldaya Martin, J. Alimena, S. Amoroso, Y. An, S. Baxter, M. Bayatmakou, H. Becerril Gonzalez, O. Behnke, A. Belvedere, S. Bhattacharya, F. Blekman, K. Borras, A. Campbell, A. Cardini, C. Cheng, F. Colombina, S. Consuegra Rodríguez, G. Correia Silva, M. De Silva, G. Eckerlin, D. Eckstein, L. I. Estevez Banos, O. Filatov, E. Gallo, A. Geiser, A. Giraldi, G. Greau, V. Guglielmi, M. Guthoff, A. Hinzmann, A. Jafari, L. Jeppe, N. Z. Jomhari, B. Kaech, M. Kasemann, C. Kleinwort, R. Kogler, M. Komm, D. Krücker, W. Lange, D. Leyva Pernia, K. Lipka, W. Lohmann, R. Mankel, I.-A. Melzer-Pellmann, M. Mendizabal Morentin, A. B. Meyer, G. Milella, A. Mussgiller, L. P. Nair, A. Nürnberg, Y. Otarid, J. Park, D. Pérez Adán, E. Ranken, A. Raspereza, B. Ribeiro Lopes, J. Rübenach, A. Saggio, M. Scham, S. Schnake, P. Schütze, C. Schwanenberger, D. Selivanova, K. Sharko, M. Shchedrolosiev, R. E. Sosa Ricardo, D. Stafford, F. Vazzoler, A. Ventura Barroso, R. Walsh, Q. Wang, Y. Wen, K. Wichmann, L. Wiens, C. Wissing, Y. Yang, A. Zimermmane Castro Santos, A. Albrecht, S. Albrecht, M. Antonello, S. Bein, L. Benato, M. Bonanomi, P. Connor, M. Eich, K. El Morabit, Y. Fischer, A. Fröhlich, C. Garbers, E. Garutti, A. Grohsjean, M. Hajheidari, J. Haller, H. R. Jabusch, G. Kasieczka, P. Keicher, R. Klanner, W. Korcari, T. Kramer, V. Kutzner, F. Labe, J. Lange, A. Lobanov, C. Matthies, A. Mehta, L. Moureaux, M. Mrowietz, A. Nigamova, Y. Nissan, A. Paasch, K. J. Pena Rodriguez, T. Quadfasel, B. Raciti, M. Rieger, D. Savoiu, J. Schindler, P. Schleper, M. Schröder, J. Schwandt, M. Sommerhalder, H. Stadie, G. Steinbrück, A. Tews, M. Wolf, S. Brommer, M. Burkart, E. Butz, T. Chwalek, A. Dierlamm, A. Droll, N. Faltermann, M. Giffels, A. Gottmann, F. Hartmann, R. Hofsaess, M. Horzela, U. Husemann, J. Kieseler, M. Klute, R. Koppenhöfer, J. M. Lawhorn, M. Link, A. Lintuluoto, S. Maier, S. Mitra, M. Mormile, Th. Müller, M. Neukum, M. Oh, M. Presilla, G. Quast, K. Rabbertz, B. Regnery, N. Shadskiy, I. Shvetsov, H. J. Simonis, M. Toms, N. Trevisani, R. Ulrich, R. F. Von Cube, M. Wassmer, S. Wieland, F. Wittig, R. Wolf, X. Zuo, G. Anagnostou, G. Daskalakis, A. Kyriakis, A. Papadopoulos, A. Stakia, P. Kontaxakis, G. Melachroinos, A. Panagiotou, I. Papavergou, I. Paraskevas, N. Saoulidou, K. Theofilatos, E. Tziaferi, K. Vellidis, I. Zisopoulos, G. Bakas, T. Chatzistavrou, G. Karapostoli, K. Kousouris, I. Papakrivopoulos, E. Siamarkou, G. Tsipolitis, A. Zacharopoulou, K. Adamidis, I. Bestintzanos, I. Evangelou, C. Foudas, P. Gianneios, C. Kamtsikis, P. Katsoulis, P. Kokkas, P. G. Kosmoglou Kioseoglou, N. Manthos, I. Papadopoulos, J. Strologas, M. Bartók, C. Hajdu, D. Horvath, F. Sikler, V. Veszpremi, M. Csanád, K. Farkas, M. M. A. Gadallah, Á. Kadlecsik, P. Major, K. Mandal, G. Pásztor, A. J. Rádl, G. I. Veres, P. Raics, B. Ujvari, G. Zilizi, G. Bencze, S. Czellar, J. Molnar, Z. Szillasi, T. Csorgo, F. Nemes, T. Novak, J. Babbar, S. Bansal, S. B. Beri, V. Bhatnagar, G. Chaudhary, S. Chauhan, N. Dhingra, A. Kaur, A. Kaur, H. Kaur, M. Kaur, S. Kumar, K. Sandeep, T. Sheokand, J. B. Singh, A. Singla, A. Ahmed, A. Bhardwaj, A. Chhetri, B. C. Choudhary, A. Kumar, A. Kumar, M. Naimuddin, K. Ranjan, S. Saumya, S. Baradia, S. Barman, S. Bhattacharya, S. Dutta, S. Dutta, P. Palit, S. Sarkar, M. M. Ameen, P. K. Behera, S. C. Behera, S. Chatterjee, P. Jana, P. Kalbhor, J. R. Komaragiri, D. Kumar, L. Panwar, P. R. Pujahari, N. R. Saha, A. Sharma, A. K. Sikdar, S. Verma, S. Dugad, M. Kumar, G. B. Mohanty, P. Suryadevara, A. Bala, S. Banerjee, R. M. Chatterjee, R. K. Dewanjee, M. Guchait, Sh. Jain, S. Karmakar, S. Kumar, G. Majumder, K. Mazumdar, S. Parolia, A. Thachayath, S. Bahinipati, C. Kar, D. Maity, P. Mal, T. Mishra, V. K. Muraleedharan Nair Bindhu, K. Naskar, A. Nayak, P. Sadangi, P. Saha, S. K. Swain, S. Varghese, D. Vats, S. Acharya, A. Alpana, S. Dube, B. Gomber, B. Kansal, A. Laha, B. Sahu, S. Sharma, H. Bakhshiansohi, E. Khazaie, M. Zeinali, S. Chenarani, S. M. Etesami, M. Khakzad, M. Mohammadi Najafabadi, M. Grunewald, M. Abbrescia, R. Aly, A. Colaleo, D. Creanza, B. D’Anzi, N. De Filippis, M. De Palma, A. Di Florio, W. Elmetenawee, L. Fiore, G. Iaselli, M. Louka, G. Maggi, M. Maggi, I. Margjeka, V. Mastrapasqua, S. My, S. Nuzzo, A. Pellecchia, A. Pompili, G. Pugliese, R. Radogna, G. Ramirez-Sanchez, D. Ramos, A. Ranieri, L. Silvestris, F. M. Simone, Ü. Sözbilir, A. Stamerra, R. Venditti, P. Verwilligen, A. Zaza, G. Abbiendi, C. Battilana, D. Bonacorsi, L. Borgonovi, R. Campanini, P. Capiluppi, A. Castro, F. R. Cavallo, M. Cuffiani, G. M. Dallavalle, T. Diotalevi, F. Fabbri, A. Fanfani, D. Fasanella, P. Giacomelli, L. Giommi, C. Grandi, L. Guiducci, S. Lo Meo, L. Lunerti, S. Marcellini, G. Masetti, F. L. Navarria, A. Perrotta, F. Primavera, A. M. Rossi, T. Rovelli, S. Costa, A. Di Mattia, R. Potenza, A. Tricomi, C. Tuve, P. Assiouras, G. Barbagli, G. Bardelli, B. Camaiani, A. Cassese, R. Ceccarelli, V. Ciulli, C. Civinini, R. D’Alessandro, E. Focardi, T. Kello, G. Latino, P. Lenzi, M. Lizzo, M. Meschini, S. Paoletti, A. Papanastassiou, G. Sguazzoni, L. Viliani, L. Benussi, S. Bianco, S. Meola, D. Piccolo, P. Chatagnon, F. Ferro, E. Robutti, S. Tosi, A. Benaglia, G. Boldrini, F. Brivio, F. Cetorelli, F. De Guio, M. E. Dinardo, P. Dini, S. Gennai, R. Gerosa, A. Ghezzi, P. Govoni, L. Guzzi, M. T. Lucchini, M. Malberti, S. Malvezzi, A. Massironi, D. Menasce, L. Moroni, M. Paganoni, D. Pedrini, B. S. Pinolini, S. Ragazzi, T. Tabarelli de Fatis, D. Zuolo, S. Buontempo, A. Cagnotta, F. Carnevali, N. Cavallo, A. De Iorio, F. Fabozzi, A. O. M. Iorio, L. Lista, P. Paolucci, B. Rossi, C. Sciacca, R. Ardino, P. Azzi, N. Bacchetta, M. Bellato, D. Bisello, P. Bortignon, A. Bragagnolo, R. Carlin, P. Checchia, T. Dorigo, F. Gasparini, U. Gasparini, E. Lusiani, M. Margoni, F. Marini, M. Migliorini, J. Pazzini, P. Ronchese, R. Rossin, F. Simonetto, G. Strong, M. Tosi, A. Triossi, S. Ventura, H. Yarar, M. Zanetti, P. Zotto, A. Zucchetta, G. Zumerle, S. Abu Zeid, C. Aimè, A. Braghieri, S. Calzaferri, D. Fiorina, P. Montagna, V. Re, C. Riccardi, P. Salvini, I. Vai, P. Vitulo, S. Ajmal, P. Asenov, G. M. Bilei, D. Ciangottini, L. Fanò, M. Magherini, G. Mantovani, V. Mariani, M. Menichelli, F. Moscatelli, A. Rossi, A. Santocchia, D. Spiga, T. Tedeschi, P. Azzurri, G. Bagliesi, R. Bhattacharya, L. Bianchini, T. Boccali, E. Bossini, D. Bruschini, R. Castaldi, M. A. Ciocci, M. Cipriani, V. D’Amante, R. Dell’Orso, S. Donato, A. Giassi, F. Ligabue, D. Matos Figueiredo, A. Messineo, M. Musich, F. Palla, A. Rizzi, G. Rolandi, S. Roy Chowdhury, T. Sarkar, A. Scribano, P. Spagnolo, R. Tenchini, G. Tonelli, N. Turini, A. Venturi, P. G. Verdini, P. Barria, M. Campana, F. Cavallari, L. Cunqueiro Mendez, D. Del Re, E. Di Marco, M. Diemoz, F. Errico, E. Longo, P. Meridiani, J. Mijuskovic, G. Organtini, F. Pandolfi, R. Paramatti, C. Quaranta, S. Rahatlou, C. Rovelli, F. Santanastasio, L. Soffi, N. Amapane, R. Arcidiacono, S. Argiro, M. Arneodo, N. Bartosik, R. Bellan, A. Bellora, C. Biino, N. Cartiglia, M. Costa, R. Covarelli, N. Demaria, L. Finco, M. Grippo, B. Kiani, F. Legger, F. Luongo, C. Mariotti, S. Maselli, A. Mecca, E. Migliore, M. Monteno, R. Mulargia, M. M. Obertino, G. Ortona, L. Pacher, N. Pastrone, M. Pelliccioni, M. Ruspa, F. Siviero, V. Sola, A. Solano, A. Staiano, C. Tarricone, D. Trocino, G. Umoret, E. Vlasov, S. Belforte, V. Candelise, M. Casarsa, F. Cossutti, K. De Leo, G. Della Ricca, S. Dogra, J. Hong, C. Huh, B. Kim, D. H. Kim, J. Kim, H. Lee, S. W. Lee, C. S. Moon, Y. D. Oh, M. S. Ryu, S. Sekmen, Y. C. Yang, M. S. Kim, G. Bak, P. Gwak, H. Kim, D. H. Moon, E. Asilar, D. Kim, T. J. Kim, J. A. Merlin, S. Choi, S. Han, B. Hong, K. Lee, K. S. Lee, S. Lee, J. Park, S. K. Park, J. Yoo, J. Goh, S. Yang, H. S. Kim, Y. Kim, S. Lee, J. Almond, J. H. Bhyun, J. Choi, W. Jun, J. Kim, S. Ko, H. Kwon, H. Lee, J. Lee, J. Lee, B. H. Oh, S. B. Oh, H. Seo, U. K. Yang, I. Yoon, W. Jang, D. Y. Kang, Y. Kang, S. Kim, B. Ko, J. S. H. Lee, Y. Lee, I. C. Park, Y. Roh, I. J. Watson, S. Ha, H. D. Yoo, M. Choi, M. R. Kim, H. Lee, Y. Lee, I. Yu, T. Beyrouthy, Y. Maghrbi, K. Dreimanis, A. Gaile, G. Pikurs, A. Potrebko, M. Seidel, V. Veckalns, N. R. Strautnieks, M. Ambrozas, A. Juodagalvis, A. Rinkevicius, G. Tamulaitis, N. Bin Norjoharuddeen, I. Yusuff, Z. Zolkapli, J. F. Benitez, A. Castaneda Hernandez, H. A. Encinas Acosta, L. G. Gallegos Maríñez, M. León Coello, J. A. Murillo Quijada, A. Sehrawat, L. Valencia Palomo, G. Ayala, H. Castilla-Valdez, E. De La Cruz-Burelo, I. Heredia-De La Cruz, R. Lopez-Fernandez, C. A. Mondragon Herrera, A. Sánchez Hernández, C. Oropeza Barrera, M. Ramírez García, I. Bautista, I. Pedraza, H. A. Salazar Ibarguen, C. Uribe Estrada, I. Bubanja, N. Raicevic, P. H. Butler, A. Ahmad, M. I. Asghar, A. Awais, M. I. M. Awan, H. R. Hoorani, W. A. Khan, V. Avati, L. Grzanka, M. Malawski, H. Bialkowska, M. Bluj, B. Boimska, M. Górski, M. Kazana, M. Szleper, P. Zalewski, K. Bunkowski, K. Doroba, A. Kalinowski, M. Konecki, J. Krolikowski, A. Muhammad, K. Pozniak, W. Zabolotny, M. Araujo, D. Bastos, C. Beirão Da Cruz E Silva, A. Boletti, M. Bozzo, T. Camporesi, G. Da Molin, P. Faccioli, M. Gallinaro, J. Hollar, N. Leonardo, T. Niknejad, A. Petrilli, M. Pisano, J. Seixas, J. Varela, J. W. Wulff, P. Adzic, P. Milenovic, M. Dordevic, J. Milosevic, V. Rekovic, M. Aguilar-Benitez, J. Alcaraz Maestre, Cristina F. Bedoya, M. Cepeda, M. Cerrada, N. Colino, B. De La Cruz, A. Delgado Peris, A. Escalante Del Valle, D. Fernández Del Val, J. P. Fernández Ramos, J. Flix, M. C. Fouz, O. Gonzalez Lopez, S. Goy Lopez, J. M. Hernandez, M. I. Josa, D. Moran, C. M. Morcillo Perez, Á. Navarro Tobar, C. Perez Dengra, A. Pérez-Calero Yzquierdo, J. Puerta Pelayo, I. Redondo, D. D. Redondo Ferrero, L. Romero, S. Sánchez Navas, L. Urda Gómez, J. Vazquez Escobar, C. Willmott, J. F. de Trocóniz, B. Alvarez Gonzalez, J. Cuevas, J. Fernandez Menendez, S. Folgueras, I. Gonzalez Caballero, J. R. González Fernández, E. Palencia Cortezon, C. Ramón Álvarez, V. Rodríguez Bouza, A. Soto Rodríguez, A. Trapote, C. Vico Villalba, P. Vischia, S. Bhowmik, S. Blanco Fernández, J. A. Brochero Cifuentes, I. J. Cabrillo, A. Calderon, J. Duarte Campderros, M. Fernandez, G. Gomez, C. Lasaosa García, C. Martinez Rivero, P. Martinez Ruiz del Arbol, F. Matorras, P. Matorras Cuevas, E. Navarrete Ramos, J. Piedra Gomez, L. Scodellaro, I. Vila, J. M. Vizan Garcia, M. K. Jayananda, B. Kailasapathy, D. U. J. Sonnadara, D. D. C. Wickramarathna, W. G. D. Dharmaratna, K. Liyanage, N. Perera, N. Wickramage, D. Abbaneo, C. Amendola, E. Auffray, G. Auzinger, J. Baechler, D. Barney, A. Bermúdez Martínez, M. Bianco, B. Bilin, A. A. Bin Anuar, A. Bocci, C. Botta, E. Brondolin, C. Caillol, G. Cerminara, N. Chernyavskaya, D. d’Enterria, A. Dabrowski, A. David, A. De Roeck, M. M. Defranchis, M. Deile, M. Dobson, L. Forthomme, G. Franzoni, W. Funk, S. Giani, D. Gigi, K. Gill, F. Glege, L. Gouskos, M. Haranko, J. Hegeman, B. Huber, V. Innocente, T. James, P. Janot, S. Laurila, P. Lecoq, E. Leutgeb, C. Lourenço, B. Maier, L. Malgeri, M. Mannelli, A. C. Marini, M. Matthewman, F. Meijers, S. Mersi, E. Meschi, V. Milosevic, F. Monti, F. Moortgat, M. Mulders, I. Neutelings, S. Orfanelli, F. Pantaleo, G. Petrucciani, A. Pfeiffer, M. Pierini, D. Piparo, H. Qu, D. Rabady, G. Reales Gutiérrez, M. Rovere, H. Sakulin, S. Scarfi, C. Schwick, M. Selvaggi, A. Sharma, K. Shchelina, P. Silva, P. Sphicas, A. G. Stahl Leiton, A. Steen, S. Summers, D. Treille, P. Tropea, A. Tsirou, D. Walter, J. Wanczyk, J. Wang, S. Wuchterl, P. Zehetner, P. Zejdl, W. D. Zeuner, T. Bevilacqua, L. Caminada, A. Ebrahimi, W. Erdmann, R. Horisberger, Q. Ingram, H. C. Kaestli, D. Kotlinski, C. Lange, M. Missiroli, L. Noehte, T. Rohe, T. K. Aarrestad, K. Androsov, M. Backhaus, A. Calandri, C. Cazzaniga, K. Datta, A. De Cosa, G. Dissertori, M. Dittmar, M. Donegà, F. Eble, M. Galli, K. Gedia, F. Glessgen, C. Grab, D. Hits, W. Lustermann, A.-M. Lyon, R. A. Manzoni, M. Marchegiani, L. Marchese, C. Martin Perez, A. Mascellani, F. Nessi-Tedaldi, F. Pauss, V. Perovic, S. Pigazzini, C. Reissel, T. Reitenspiess, B. Ristic, F. Riti, D. Ruini, R. Seidita, J. Steggemann, D. Valsecchi, R. Wallny, C. Amsler, P. Bärtschi, D. Brzhechko, M. F. Canelli, K. Cormier, J. K. Heikkilä, M. Huwiler, W. Jin, A. Jofrehei, B. Kilminster, S. Leontsinis, S. P. Liechti, A. Macchiolo, P. Meiring, U. Molinatti, A. Reimers, P. Robmann, S. Sanchez Cruz, M. Senger, Y. Takahashi, R. Tramontano, C. Adloff, D. Bhowmik, C. M. Kuo, W. Lin, P. K. Rout, P. C. Tiwari, S. S. Yu, L. Ceard, Y. Chao, K. F. Chen, P. s. Chen, Z. g. Chen, W. -S. Hou, T. h. Hsu, Y. w. Kao, R. Khurana, G. Kole, Y. y. Li, R.-S. Lu, E. Paganis, X. f. Su, J. Thomas-Wilsker, L. s. Tsai, H. y. Wu, E. Yazgan, C. Asawatangtrakuldee, N. Srimanobhas, V. Wachirapusitanand, D. Agyel, F. Boran, Z. S. Demiroglu, F. Dolek, I. Dumanoglu, E. Eskut, Y. Guler, E. Gurpinar Guler, C. Isik, O. Kara, A. Kayis Topaksu, U. Kiminsu, G. Onengut, K. Ozdemir, A. Polatoz, B. Tali, U. G. Tok, S. Turkcapar, E. Uslan, I. S. Zorbakir, M. Yalvac, B. Akgun, I. O. Atakisi, E. Gülmez, M. Kaya, O. Kaya, S. Tekten, A. Cakir, K. Cankocak, Y. Komurcu, S. Sen, O. Aydilek, S. Cerci, V. Epshteyn, B. Hacisahinoglu, I. Hos, B. Kaynak, S. Ozkorucuklu, O. Potok, H. Sert, C. Simsek, C. Zorbilmez, B. Isildak, D. Sunar Cerci, A. Boyaryntsev, B. Grynyov, L. Levchuk, D. Anthony, J. J. Brooke, A. Bundock, F. Bury, E. Clement, D. Cussans, H. Flacher, M. Glowacki, J. Goldstein, H. F. Heath, L. Kreczko, S. Paramesvaran, S. Seif El Nasr-Storey, V. J. Smith, N. Stylianou, K. Walkingshaw Pass, R. White, A. H. Ball, K. W. Bell, A. Belyaev, C. Brew, R. M. Brown, D. J. A. Cockerill, C. Cooke, K. V. Ellis, K. Harder, S. Harper, M.-L. Holmberg, J. Linacre, K. Manolopoulos, D. M. Newbold, E. Olaiya, D. Petyt, T. Reis, G. Salvi, T. Schuh, C. H. Shepherd-Themistocleous, I. R. Tomalin, T. Williams, R. Bainbridge, P. Bloch, C. E. Brown, O. Buchmuller, V. Cacchio, C. A. Carrillo Montoya, G. S. Chahal, D. Colling, J. S. Dancu, I. Das, P. Dauncey, G. Davies, J. Davies, M. Della Negra, S. Fayer, G. Fedi, G. Hall, M. H. Hassanshahi, A. Howard, G. Iles, M. Knight, J. Langford, J. León Holgado, L. Lyons, A.-M. Magnan, S. Malik, M. Mieskolainen, J. Nash, M. Pesaresi, B. C. Radburn-Smith, A. Richards, A. Rose, C. Seez, R. Shukla, A. Tapper, K. Uchida, G. P. Uttley, L. H. Vage, T. Virdee, M. Vojinovic, N. Wardle, D. Winterbottom, K. Coldham, J. E. Cole, A. Khan, P. Kyberd, I. D. Reid, S. Abdullin, A. Brinkerhoff, B. Caraway, J. Dittmann, K. Hatakeyama, J. Hiltbrand, B. McMaster, M. Saunders, S. Sawant, C. Sutantawibul, J. Wilson, R. Bartek, A. Dominguez, C. Huerta Escamilla, A. E. Simsek, R. Uniyal, A. M. Vargas Hernandez, B. Bam, R. Chudasama, S. I. Cooper, S. V. Gleyzer, C. U. Perez, P. Rumerio, E. Usai, R. Yi, A. Akpinar, D. Arcaro, C. Cosby, Z. Demiragli, C. Erice, C. Fangmeier, C. Fernandez Madrazo, E. Fontanesi, D. Gastler, F. Golf, S. Jeon, I. Reed, J. Rohlf, K. Salyer, D. Sperka, D. Spitzbart, I. Suarez, A. Tsatsos, S. Yuan, A. G. Zecchinelli, G. Benelli, X. Coubez, D. Cutts, M. Hadley, U. Heintz, J. M. Hogan, T. Kwon, G. Landsberg, K. T. Lau, D. Li, J. Luo, S. Mondal, M. Narain, N. Pervan, S. Sagir, F. Simpson, M. Stamenkovic, W. Y. Wong, X. Yan, W. Zhang, S. Abbott, J. Bonilla, C. Brainerd, R. Breedon, M. Calderon De La Barca Sanchez, M. Chertok, M. Citron, J. Conway, P. T. Cox, R. Erbacher, F. Jensen, O. Kukral, G. Mocellin, M. Mulhearn, D. Pellett, W. Wei, Y. Yao, F. Zhang, M. Bachtis, R. Cousins, A. Datta, G. Flores Avila, J. Hauser, M. Ignatenko, M. A. Iqbal, T. Lam, E. Manca, A. Nunez Del Prado, D. Saltzberg, V. Valuev, R. Clare, J. W. Gary, M. Gordon, G. Hanson, W. Si, S. Wimpenny, J. G. Branson, S. Cittolin, S. Cooperstein, D. Diaz, J. Duarte, L. Giannini, J. Guiang, R. Kansal, V. Krutelyov, R. Lee, J. Letts, M. Masciovecchio, F. Mokhtar, S. Mukherjee, M. Pieri, M. Quinnan, B. V. Sathia Narayanan, V. Sharma, M. Tadel, E. Vourliotis, F. Würthwein, Y. Xiang, A. Yagil, A. Barzdukas, L. Brennan, C. Campagnari, A. Dorsett, J. Incandela, J. Kim, A. J. Li, P. Masterson, H. Mei, J. Richman, U. Sarica, R. Schmitz, F. Setti, J. Sheplock, D. Stuart, T. Á. Vámi, S. Wang, A. Bornheim, O. Cerri, A. Latorre, J. Mao, H. B. Newman, M. Spiropulu, J. R. Vlimant, C. Wang, S. Xie, R. Y. Zhu, J. Alison, S. An, M. B. Andrews, P. Bryant, M. Cremonesi, V. Dutta, T. Ferguson, A. Harilal, C. Liu, T. Mudholkar, S. Murthy, M. Paulini, A. Roberts, A. Sanchez, W. Terrill, J. P. Cumalat, W.T. Ford, A. Hassani, G. Karathanasis, E. MacDonald, N. Manganelli, A. Perloff, C. Savard, N. Schonbeck, K. Stenson, K. A. Ulmer, S. R. Wagner, N. Zipper, J. Alexander, S. Bright-Thonney, X. Chen, D. J. Cranshaw, J. Fan, X. Fan, D. Gadkari, S. Hogan, P. Kotamnives, J. Monroy, M. Oshiro, J. R. Patterson, J. Reichert, M. Reid, A. Ryd, J. Thom, P. Wittich, R. Zou, M. Albrow, M. Alyari, O. Amram, G. Apollinari, A. Apresyan, L. A. T. Bauerdick, D. Berry, J. Berryhill, P. C. Bhat, K. Burkett, J. N. Butler, A. Canepa, G. B. Cerati, H. W. K. Cheung, F. Chlebana, G. Cummings, J. Dickinson, I. Dutta, V. D. Elvira, Y. Feng, J. Freeman, A. Gandrakota, Z. Gecse, L. Gray, D. Green, A. Grummer, S. Grünendahl, D. Guerrero, O. Gutsche, R. M. Harris, R. Heller, T. C. Herwig, J. Hirschauer, L. Horyn, B. Jayatilaka, S. Jindariani, M. Johnson, U. Joshi, T. Klijnsma, B. Klima, K. H. M. Kwok, S. Lammel, D. Lincoln, R. Lipton, T. Liu, C. Madrid, K. Maeshima, C. Mantilla, D. Mason, P. McBride, P. Merkel, S. Mrenna, S. Nahn, J. Ngadiuba, D. Noonan, V. Papadimitriou, N. Pastika, K. Pedro, C. Pena, F. Ravera, A. Reinsvold Hall, L. Ristori, E. Sexton-Kennedy, N. Smith, A. Soha, L. Spiegel, S. Stoynev, J. Strait, L. Taylor, S. Tkaczyk, N. V. Tran, L. Uplegger, E. W. Vaandering, I. Zoi, C. Aruta, P. Avery, D. Bourilkov, L. Cadamuro, P. Chang, V. Cherepanov, R. D. Field, E. Koenig, M. Kolosova, J. Konigsberg, A. Korytov, K. H. Lo, K. Matchev, N. Menendez, G. Mitselmakher, K. Mohrman, A. Muthirakalayil Madhu, N. Rawal, D. Rosenzweig, S. Rosenzweig, K. Shi, J. Wang, T. Adams, A. Al Kadhim, A. Askew, N. Bower, R. Habibullah, V. Hagopian, R. Hashmi, R. S. Kim, S. Kim, T. Kolberg, G. Martinez, H. Prosper, P. R. Prova, M. Wulansatiti, R. Yohay, J. Zhang, B. Alsufyani, M. M. Baarmand, S. Butalla, T. Elkafrawy, M. Hohlmann, R. Kumar Verma, M. Rahmani, E. Yanes, M. R. Adams, A. Baty, C. Bennett, R. Cavanaugh, R. Escobar Franco, O. Evdokimov, C. E. Gerber, D. J. Hofman, J. h. Lee, D. S. Lemos, A. H. Merrit, C. Mills, S. Nanda, G. Oh, B. Ozek, D. Pilipovic, R. Pradhan, T. Roy, S. Rudrabhatla, M. B. Tonjes, N. Varelas, Z. Ye, J. Yoo, M. Alhusseini, D. Blend, K. Dilsiz, L. Emediato, G. Karaman, O. K. Köseyan, J.-P. Merlo, A. Mestvirishvili, J. Nachtman, O. Neogi, H. Ogul, Y. Onel, A. Penzo, C. Snyder, E. Tiras, B. Blumenfeld, L. Corcodilos, J. Davis, A. V. Gritsan, L. Kang, S. Kyriacou, P. Maksimovic, M. Roguljic, J. Roskes, S. Sekhar, M. Swartz, A. Abreu, L. F. Alcerro Alcerro, J. Anguiano, P. Baringer, A. Bean, Z. Flowers, D. Grove, J. King, G. Krintiras, M. Lazarovits, C. Le Mahieu, C. Lindsey, J. Marquez, N. Minafra, M. Murray, M. Nickel, M. Pitt, S. Popescu, C. Rogan, C. Royon, R. Salvatico, S. Sanders, C. Smith, Q. Wang, G. Wilson, B. Allmond, A. Ivanov, K. Kaadze, A. Kalogeropoulos, D. Kim, Y. Maravin, K. Nam, J. Natoli, D. Roy, G. Sorrentino, F. Rebassoo, D. Wright, A. Baden, A. Belloni, Y. M. Chen, S. C. Eno, N. J. Hadley, S. Jabeen, R. G. Kellogg, T. Koeth, Y. Lai, S. Lascio, A. C. Mignerey, S. Nabili, C. Palmer, C. Papageorgakis, M. M. Paranjpe, L. Wang, J. Bendavid, W. Busza, I. A. Cali, M. D’Alfonso, J. Eysermans, C. Freer, G. Gomez-Ceballos, M. Goncharov, G. Grosso, P. Harris, D. Hoang, D. Kovalskyi, J. Krupa, L. Lavezzo, Y.-J. Lee, K. Long, C. Mironov, C. Paus, D. Rankin, C. Roland, G. Roland, S. Rothman, G. S. F. Stephans, Z. Wang, B. Wyslouch, T. J. Yang, B. Crossman, B. M. Joshi, C. Kapsiak, M. Krohn, D. Mahon, J. Mans, B. Marzocchi, S. Pandey, M. Revering, R. Rusack, R. Saradhy, N. Schroeder, N. Strobbe, M. A. Wadud, L. M. Cremaldi, K. Bloom, D. R. Claes, G. Haza, J. Hossain, C. Joo, I. Kravchenko, J. E. Siado, W. Tabb, A. Vagnerini, A. Wightman, F. Yan, D. Yu, H. Bandyopadhyay, L. Hay, I. Iashvili, A. Kharchilava, M. Morris, D. Nguyen, S. Rappoccio, H. Rejeb Sfar, A. Williams, G. Alverson, E. Barberis, J. Dervan, Y. Haddad, Y. Han, A. Krishna, J. Li, M. Lu, G. Madigan, R. Mccarthy, D. M. Morse, V. Nguyen, T. Orimoto, A. Parker, L. Skinnari, A. Tishelman-Charny, B. Wang, D. Wood, S. Bhattacharya, J. Bueghly, Z. Chen, S. Dittmer, K. A. Hahn, Y. Liu, Y. Miao, D. G. Monk, M. H. Schmitt, A. Taliercio, M. Velasco, G. Agarwal, R. Band, R. Bucci, S. Castells, A. Das, R. Goldouzian, M. Hildreth, K. W. Ho, K. Hurtado Anampa, T. Ivanov, C. Jessop, K. Lannon, J. Lawrence, N. Loukas, L. Lutton, J. Mariano, N. Marinelli, I. Mcalister, T. McCauley, C. Mcgrady, C. Moore, Y. Musienko, H. Nelson, M. Osherson, A. Piccinelli, R. Ruchti, A. Townsend, Y. Wan, M. Wayne, H. Yockey, M. Zarucki, L. Zygala, A. Basnet, B. Bylsma, M. Carrigan, L.S. Durkin, C. Hill, M. Joyce, M. Nunez Ornelas, K. Wei, B. L. Winer, B. R. Yates, F. M. Addesa, H. Bouchamaoui, P. Das, G. Dezoort, P. Elmer, A. Frankenthal, B. Greenberg, N. Haubrich, G. Kopp, S. Kwan, D. Lange, A. Loeliger, D. Marlow, I. Ojalvo, J. Olsen, A. Shevelev, D. Stickland, C. Tully, S. Malik, A. S. Bakshi, V. E. Barnes, S. Chandra, R. Chawla, S. Das, A. Gu, L. Gutay, M. Jones, A. W. Jung, D. Kondratyev, A. M. Koshy, M. Liu, G. Negro, N. Neumeister, G. Paspalaki, S. Piperov, V. Scheurer, J. F. Schulte, M. Stojanovic, J. Thieman, A. K. Virdi, F. Wang, W. Xie, J. Dolen, N. Parashar, A. Pathak, D. Acosta, T. Carnahan, K. M. Ecklund, P. J. Fernández Manteca, S. Freed, P. Gardner, F. J. M. Geurts, W. Li, O. Miguel Colin, B. P. Padley, R. Redjimi, J. Rotter, E. Yigitbasi, Y. Zhang, A. Bodek, P. de Barbaro, R. Demina, J. L. Dulemba, A. Garcia-Bellido, O. Hindrichs, A. Khukhunaishvili, N. Parmar, P. Parygin, E. Popova, R. Taus, K. Goulianos, B. Chiarito, J. P. Chou, Y. Gershtein, E. Halkiadakis, A. Hart, M. Heindl, D. Jaroslawski, O. Karacheban, I. Laflotte, A. Lath, R. Montalvo, K. Nash, H. Routray, S. Salur, S. Schnetzer, S. Somalwar, R. Stone, S. A. Thayil, S. Thomas, J. Vora, H. Wang, H. Acharya, D. Ally, A. G. Delannoy, S. Fiorendi, S. Higginbotham, T. Holmes, A. R. Kanuganti, N. Karunarathna, L. Lee, E. Nibigira, S. Spanier, D. Aebi, M. Ahmad, O. Bouhali, R. Eusebi, J. Gilmore, T. Huang, T. Kamon, H. Kim, S. Luo, R. Mueller, D. Overton, D. Rathjens, A. Safonov, N. Akchurin, J. Damgov, V. Hegde, A. Hussain, Y. Kazhykarim, K. Lamichhane, S. W. Lee, A. Mankel, T. Peltola, I. Volobouev, A. Whitbeck, E. Appelt, Y. Chen, S. Greene, A. Gurrola, W. Johns, R. Kunnawalkam Elayavalli, A. Melo, F. Romeo, P. Sheldon, S. Tuo, J. Velkovska, J. Viinikainen, B. Cardwell, B. Cox, J. Hakala, R. Hirosky, A. Ledovskoy, C. Neu, C. E. Perez Lara, P. E. Karchin, A. Aravind, S. Banerjee, K. Black, T. Bose, S. Dasu, I. De Bruyn, P. Everaerts, C. Galloni, H. He, M. Herndon, A. Herve, C. K. Koraka, A. Lanaro, R. Loveless, J. Madhusudanan Sreekala, A. Mallampalli, A. Mohammadi, S. Mondal, G. Parida, D. Pinna, A. Savin, V. Shang, V. Sharma, W. H. Smith, D. Teague, H. F. Tsoi, W. Vetens, A. Warden, S. Afanasiev, V. Andreev, Yu. Andreev, T. Aushev, M. Azarkin, A. Babaev, A. Belyaev, V. Blinov, E. Boos, V. Borshch, D. Budkouski, M. Chadeeva, V. Chekhovsky, R. Chistov, A. Dermenev, T. Dimova, D. Druzhkin, M. Dubinin, L. Dudko, A. Ershov, G. Gavrilov, V. Gavrilov, S. Gninenko, V. Golovtcov, N. Golubev, I. Golutvin, I. Gorbunov, Y. Ivanov, V. Kachanov, V. Karjavine, A. Karneyeu, V. Kim, M. Kirakosyan, D. Kirpichnikov, M. Kirsanov, V. Klyukhin, O. Kodolova, V. Korenkov, A. Kozyrev, N. Krasnikov, A. Lanev, P. Levchenko, O. Lukina, N. Lychkovskaya, V. Makarenko, A. Malakhov, V. Matveev, V. Murzin, A. Nikitenko, S. Obraztsov, V. Oreshkin, V. Palichik, V. Perelygin, S. Petrushanko, S. Polikarpov, V. Popov, O. Radchenko, M. Savina, V. Savrin, V. Shalaev, S. Shmatov, S. Shulha, Y. Skovpen, S. Slabospitskii, V. Smirnov, A. Snigirev, D. Sosnov, V. Sulimov, E. Tcherniaev, A. Terkulov, O. Teryaev, I. Tlisova, A. Toropin, L. Uvarov, A. Uzunian, I. Vardanyan, A. Vorobyev, N. Voytishin, B. S. Yuldashev, A. Zarubin, I. Zhizhin, A. Zhokin

**Affiliations:** 1 Yerevan Physics Institute, Yerevan, Armenia; 2 Institut für Hochenergiephysik, Vienna, Austria; 3 Universiteit Antwerpen, Antwerpen, Belgium; 4 Vrije Universiteit Brussel, Brussels, Belgium; 5 Université Libre de Bruxelles, Brussels, Belgium; 6 Ghent University, Ghent, Belgium; 7 Université Catholique de Louvain, Louvain-la-Neuve, Belgium; 8 Centro Brasileiro de Pesquisas Fisicas, Rio de Janeiro, Brazil; 9 Universidade do Estado do Rio de Janeiro, Rio de Janeiro, Brazil; 10 Universidade Estadual Paulista, Universidade Federal do ABC, São Paulo, Brazil; 11 Institute for Nuclear Research and Nuclear Energy, Bulgarian Academy of Sciences, Sofia, Bulgaria; 12 University of Sofia, Sofia, Bulgaria; 13 Instituto De Alta Investigación, Universidad de Tarapacá, Casilla 7 D, Arica, Chile; 14 Beihang University, Beijing, China; 15 Department of Physics, Tsinghua University, Beijing, China; 16 Institute of High Energy Physics, Beijing, China; 17 State Key Laboratory of Nuclear Physics and Technology, Peking University, Beijing, China; 18 Sun Yat-Sen University, Guangzhou, China; 19 University of Science and Technology of China, Hefei, China; 20 Nanjing Normal University, Nanjing, China; 21 Institute of Modern Physics and Key Laboratory of Nuclear Physics and Ion-beam Application (MOE)-Fudan University, Shanghai, China; 22 Zhejiang University, Hangzhou, Zhejiang China; 23 Universidad de Los Andes, Bogotá, Colombia; 24 Universidad de Antioquia, Medellin, Colombia; 25 University of Split, Faculty of Electrical Engineering, Mechanical Engineering and Naval Architecture, Split, Croatia; 26 Faculty of Science, University of Split, Split, Croatia; 27 Institute Rudjer Boskovic, Zagreb, Croatia; 28 University of Cyprus, Nicosia, Cyprus; 29 Charles University, Prague, Czech Republic; 30 Escuela Politecnica Nacional, Quito, Ecuador; 31 Universidad San Francisco de Quito, Quito, Ecuador; 32 Academy of Scientific Research and Technology of the Arab Republic of Egypt, Egyptian Network of High Energy Physics, Cairo, Egypt; 33 Center for High Energy Physics (CHEP-FU), Fayoum University, El-Fayoum, Egypt; 34 National Institute of Chemical Physics and Biophysics, Tallinn, Estonia; 35 Department of Physics, University of Helsinki, Helsinki, Finland; 36 Helsinki Institute of Physics, Helsinki, Finland; 37 Lappeenranta-Lahti University of Technology, Lappeenranta, Finland; 38 IRFU, CEA, Université Paris-Saclay, Gif-sur-Yvette, France; 39 Laboratoire Leprince-Ringuet, CNRS/IN2P3, Ecole Polytechnique, Institut Polytechnique de Paris, Palaiseau, France; 40 CNRS, IPHC UMR 7178, Université de Strasbourg, Strasbourg, France; 41 Institut de Physique des 2 Infinis de Lyon (IP2I ), Villeurbanne, France; 42 Georgian Technical University, Tbilisi, Georgia; 43 RWTH Aachen University, I. Physikalisches Institut, Aachen, Germany; 44 RWTH Aachen University, III. Physikalisches Institut A, Aachen, Germany; 45 RWTH Aachen University, III. Physikalisches Institut B, Aachen, Germany; 46 Deutsches Elektronen-Synchrotron, Hamburg, Germany; 47 University of Hamburg, Hamburg, Germany; 48 Karlsruher Institut fuer Technologie, Karlsruhe, Germany; 49 Institute of Nuclear and Particle Physics (INPP), NCSR Demokritos, Aghia Paraskevi, Greece; 50 National and Kapodistrian University of Athens, Athens, Greece; 51 National Technical University of Athens, Athens, Greece; 52 University of Ioánnina, Ioannina, Greece; 53 HUN-REN Wigner Research Centre for Physics, Budapest, Hungary; 54 MTA-ELTE Lendület CMS Particle and Nuclear Physics Group, Eötvös Loránd University, Budapest, Hungary; 55 Faculty of Informatics, University of Debrecen, Debrecen, Hungary; 56 Institute of Nuclear Research ATOMKI, Debrecen, Hungary; 57 Karoly Robert Campus, MATE Institute of Technology, Gyongyos, Hungary; 58 Panjab University, Chandigarh, India; 59 University of Delhi, Delhi, India; 60 Saha Institute of Nuclear Physics, HBNI, Kolkata, India; 61 Indian Institute of Technology Madras, Madras, India; 62 Tata Institute of Fundamental Research-A, Mumbai, India; 63 Tata Institute of Fundamental Research-B, Mumbai, India; 64 National Institute of Science Education and Research, An OCC of Homi Bhabha National Institute, Bhubaneswar, Odisha India; 65 Indian Institute of Science Education and Research (IISER), Pune, India; 66 Isfahan University of Technology, Isfahan, Iran; 67 Institute for Research in Fundamental Sciences (IPM), Tehran, Iran; 68 University College Dublin, Dublin, Ireland; 69 INFN Sezione di Bari, Università di Bari, Politecnico di Bari, Bari, Italy; 70 INFN Sezione di Bologna, Università di Bologna, Bologna, Italy; 71 INFN Sezione di Catania, Università di Catania, Catania, Italy; 72 INFN Sezione di Firenze, Università di Firenze, Firenze, Italy; 73 INFN Laboratori Nazionali di Frascati, Frascati, Italy; 74 INFN Sezione di Genova, Università di Genova, Genoa, Italy; 75 INFN Sezione di Milano-Bicocca, Università di Milano-Bicocca, Milan, Italy; 76 INFN Sezione di Napoli, Università di Napoli ’Federico II’, Napoli, Italy; Università della Basilicata, Potenza, Italy; Scuola Superiore Meridionale (SSM), Naples, Italy; 77 INFN Sezione di Padova, Università di Padova, Padova, Italy; Università di Trento, Trento, Italy; 78 INFN Sezione di Pavia, Università di Pavia, Pavia, Italy; 79 INFN Sezione di Perugia, Università di Perugia, Perugia, Italy; 80 INFN Sezione di Pisa, Università di Pisa, Scuola Normale Superiore di Pisa, Pisa, Italy; Università di Siena, Siena, Italy; 81 INFN Sezione di Roma, Sapienza Università di Roma, Rome, Italy; 82 INFN Sezione di Torino, Università di Torino, Torino, Italy; Università del Piemonte Orientale, Novara, Italy; 83 INFN Sezione di Trieste, Università di Trieste, Trieste, Italy; 84 Kyungpook National University, Daegu, Korea; 85 Department of Mathematics and Physics-GWNU, Gangneung, Korea; 86 Chonnam National University, Institute for Universe and Elementary Particles, Kwangju, Korea; 87 Hanyang University, Seoul, Korea; 88 Korea University, Seoul, Korea; 89 Department of Physics, Kyung Hee University, Seoul, Korea; 90 Sejong University, Seoul, Korea; 91 Seoul National University, Seoul, Korea; 92 University of Seoul, Seoul, Korea; 93 Yonsei University, Department of Physics, Seoul, Korea; 94 Sungkyunkwan University, Suwon, Korea; 95 College of Engineering and Technology, American University of the Middle East (AUM), Dasman, Kuwait; 96 Riga Technical University, Riga, Latvia; 97 University of Latvia (LU), Riga, Latvia; 98 Vilnius University, Vilnius, Lithuania; 99 National Centre for Particle Physics, Universiti Malaya, Kuala Lumpur, Malaysia; 100 Universidad de Sonora (UNISON), Hermosillo, Mexico; 101 Centro de Investigacion y de Estudios Avanzados del IPN, Mexico City, Mexico; 102 Universidad Iberoamericana, Mexico City, Mexico; 103 Benemerita Universidad Autonoma de Puebla, Puebla, Mexico; 104 University of Montenegro, Podgorica, Montenegro; 105 University of Canterbury, Christchurch, New Zealand; 106 National Centre for Physics, Quaid-I-Azam University, Islamabad, Pakistan; 107 Faculty of Computer Science, Electronics and Telecommunications, AGH University of Krakow, Kraków, Poland; 108 National Centre for Nuclear Research, Swierk, Poland; 109 Institute of Experimental Physics, Faculty of Physics, University of Warsaw, Warsaw, Poland; 110 Warsaw University of Technology, Warsaw, Poland; 111 Laboratório de Instrumentação e Física Experimental de Partículas, Lisbon, Portugal; 112 Faculty of Physics, University of Belgrade, Belgrade, Serbia; 113 VINCA Institute of Nuclear Sciences, University of Belgrade, Belgrade, Serbia; 114 Centro de Investigaciones Energéticas Medioambientales y Tecnológicas (CIEMAT), Madrid, Spain; 115 Universidad Autónoma de Madrid, Madrid, Spain; 116 Universidad de Oviedo, Instituto Universitario de Ciencias y Tecnologías Espaciales de Asturias (ICTEA), Oviedo, Spain; 117 Instituto de Física de Cantabria (IFCA), CSIC-Universidad de Cantabria, Santander, Spain; 118 University of Colombo, Colombo, Sri Lanka; 119 Department of Physics, University of Ruhuna, Matara, Sri Lanka; 120 CERN, European Organization for Nuclear Research, Geneva, Switzerland; 121 Paul Scherrer Institut, Villigen, Switzerland; 122 ETH Zurich-Institute for Particle Physics and Astrophysics (IPA), Zurich, Switzerland; 123 Universität Zürich, Zurich, Switzerland; 124 National Central University, Chung-Li, Taiwan; 125 National Taiwan University (NTU), Taipei, Taiwan; 126 High Energy Physics Research Unit, Department of Physics, Faculty of Science, Chulalongkorn University, Bangkok, Thailand; 127 Çukurova University, Physics Department, Science and Art Faculty, Adana, Turkey; 128 Physics Department, Middle East Technical University, Ankara, Turkey; 129 Bogazici University, Istanbul, Turkey; 130 Istanbul Technical University, Istanbul, Turkey; 131 Istanbul University, Istanbul, Turkey; 132 Yildiz Technical University, Istanbul, Turkey; 133 Institute for Scintillation Materials of National Academy of Science of Ukraine, Kharkiv, Ukraine; 134 Kharkiv Institute of Physics and Technology, National Science Centre, Kharkiv, Ukraine; 135 University of Bristol, Bristol, UK; 136 Rutherford Appleton Laboratory, Didcot, UK; 137 Imperial College, London, UK; 138 Brunel University, Uxbridge, UK; 139 Baylor University, Waco, TX USA; 140 Catholic University of America, Washington, DC USA; 141 The University of Alabama, Tuscaloosa, AL USA; 142 Boston University, Boston, MA USA; 143 Brown University, Providence, RI USA; 144 University of California, Davis, Davis, CA USA; 145 University of California, Los Angeles, CA USA; 146 University of California, Riverside, Riverside, CA USA; 147 University of California, San Diego, La Jolla, CA USA; 148 Department of Physics, University of California, Santa Barbara, Santa Barbara, CA USA; 149 California Institute of Technology, Pasadena, CA USA; 150 Carnegie Mellon University, Pittsburgh, PA USA; 151 University of Colorado Boulder, Boulder, CO USA; 152 Cornell University, Ithaca, NY USA; 153 Fermi National Accelerator Laboratory, Batavia, IL USA; 154 University of Florida, Gainesville, FL USA; 155 Florida State University, Tallahassee, FL USA; 156 Florida Institute of Technology, Melbourne, FL USA; 157 University of Illinois Chicago, Chicago, USA; 158 The University of Iowa, Iowa City, IA USA; 159 Johns Hopkins University, Baltimore, MD USA; 160 The University of Kansas, Lawrence, KS USA; 161 Kansas State University, Manhattan, KS USA; 162 Lawrence Livermore National Laboratory, Livermore, CA USA; 163 University of Maryland, College Park, MD USA; 164 Massachusetts Institute of Technology, Cambridge, MA USA; 165 University of Minnesota, Minneapolis, MN USA; 166 University of Mississippi, Oxford, MS USA; 167 University of Nebraska-Lincoln, Lincoln, NE USA; 168 State University of New York at Buffalo, Buffalo, NY USA; 169 Northeastern University, Boston, MA USA; 170 Northwestern University, Evanston, IL USA; 171 University of Notre Dame, Notre Dame, IN USA; 172 The Ohio State University, Columbus, OH USA; 173 Princeton University, Princeton, NJ USA; 174 University of Puerto Rico, Mayaguez, PR USA; 175 Purdue University, West Lafayette, IN USA; 176 Purdue University Northwest, Hammond, IN USA; 177 Rice University, Houston, TX USA; 178 University of Rochester, Rochester, NY USA; 179 The Rockefeller University, New York, NY USA; 180 Rutgers, The State University of New Jersey, Piscataway, NJ USA; 181 University of Tennessee, Knoxville, TN USA; 182 Texas A &M University, College Station, TX USA; 183 Texas Tech University, Lubbock, TX USA; 184 Vanderbilt University, Nashville, TN USA; 185 University of Virginia, Charlottesville, VA USA; 186 Wayne State University, Detroit, MI USA; 187 University of Wisconsin-Madison, Madison, WI USA; 188 Authors Affiliated with an Institute or an International Laboratory Covered by a Cooperation Agreement with CERN, Geneva, Switzerland; 189 Yerevan State University, Yerevan, Armenia; 190 TU Wien, Vienna, Austria; 191 Institute of Basic and Applied Sciences, Faculty of Engineering, Arab Academy for Science, Technology and Maritime Transport, Alexandria, Egypt; 192 Ghent University, Ghent, Belgium; 193 Universidade Estadual de Campinas, Campinas, Brazil; 194 Federal University of Rio Grande do Sul, Porto Alegre, Brazil; 195 UFMS, Nova Andradina, Brazil; 196 Nanjing Normal University, Nanjing, China; 197 Now at The University of Iowa, Iowa City, IA USA; 198 University of Chinese Academy of Sciences, Beijing, China; 199 China Center of Advanced Science and Technology, Beijing, China; 200 University of Chinese Academy of Sciences, Beijing, China; 201 China Spallation Neutron Source, Guangdong, China; 202 Henan Normal University, Xinxiang, China; 203 Université Libre de Bruxelles, Brussels, Belgium; 204 University of Latvia (LU), Riga, Latvia; 205 An Institute or an International Laboratory Covered by a Cooperation Agreement with CERN, Geneva, Switzerland; 206 Helwan University, Cairo, Egypt; 207 Zewail City of Science and Technology, Zewail, Egypt; 208 British University in Egypt, Cairo, Egypt; 209 Ain Shams University, Cairo, Egypt; 210 Purdue University, West Lafayette, IN USA; 211 Université de Haute Alsace, Mulhouse, France; 212 Department of Physics, Tsinghua University, Beijing, China; 213 Tbilisi State University, Tbilisi, Georgia; 214 The University of the State of Amazonas, Manaus, Brazil; 215 Erzincan Binali Yildirim University, Erzincan, Turkey; 216 University of Hamburg, Hamburg, Germany; 217 RWTH Aachen University, III. Physikalisches Institut A, Aachen, Germany; 218 Isfahan University of Technology, Isfahan, Iran; 219 Bergische University Wuppertal (BUW), Wuppertal, Germany; 220 Brandenburg University of Technology, Cottbus, Germany; 221 Forschungszentrum Jülich, Juelich, Germany; 222 CERN, European Organization for Nuclear Research, Geneva, Switzerland; 223 An Institute or an International Laboratory Covered by a Cooperation Agreement with CERN, Geneva, Switzerland; 224 Institute of Physics, University of Debrecen, Debrecen, Hungary; 225 Institute of Nuclear Research ATOMKI, Debrecen, Hungary; 226 Universitatea Babes-Bolyai-Facultatea de Fizica, Cluj-Napoca, Romania; 227 Physics Department, Faculty of Science, Assiut University, Assiut, Egypt; 228 HUN-REN Wigner Research Centre for Physics, Budapest, Hungary; 229 Punjab Agricultural University, Ludhiana, India; 230 University of Visva-Bharati, Santiniketan, India; 231 Indian Institute of Science (IISc), Bangalore, India; 232 Birla Institute of Technology, Mesra, Mesra, India; 233 IIT Bhubaneswar, Bhubaneswar, India; 234 Institute of Physics, Bhubaneswar, India; 235 University of Hyderabad, Hyderabad, India; 236 Deutsches Elektronen-Synchrotron, Hamburg, Germany; 237 Department of Physics, Isfahan University of Technology, Isfahan, Iran; 238 Sharif University of Technology, Tehran, Iran; 239 Department of Physics, University of Science and Technology of Mazandaran, Behshahr, Iran; 240 Italian National Agency for New Technologies, Energy and Sustainable Economic Development, Bologna, Italy; 241 Centro Siciliano di Fisica Nucleare e di Struttura Della Materia, Catania, Italy; 242 Università degli Studi Guglielmo Marconi, Rome, Italy; 243 Scuola Superiore Meridionale, Università di Napoli ’Federico II’, Naples, Italy; 244 Fermi National Accelerator Laboratory, Batavia, IL USA; 245 Consiglio Nazionale delle Ricerche-Istituto Officina dei Materiali, Perugia, Italy; 246 Riga Technical University, Riga, Latvia; 247 Department of Applied Physics, Faculty of Science and Technology, Universiti Kebangsaan Malaysia, Bangi, Malaysia; 248 Consejo Nacional de Ciencia y Tecnología, Mexico City, Mexico; 249 Trincomalee Campus, Eastern University, Sri Lanka, Nilaveli, Sri Lanka; 250 Saegis Campus, Nugegoda, Sri Lanka; 251 National and Kapodistrian University of Athens, Athens, Greece; 252 Ecole Polytechnique Fédérale Lausanne, Lausanne, Switzerland; 253 Universität Zürich, Zurich, Switzerland; 254 Stefan Meyer Institute for Subatomic Physics, Vienna, Austria; 255 Laboratoire d’Annecy-le-Vieux de Physique des Particules, IN2P3-CNRS, Annecy-le-Vieux, France; 256 Near East University, Research Center of Experimental Health Science, Mersin, Turkey; 257 Konya Technical University, Konya, Turkey; 258 Izmir Bakircay University, Izmir, Turkey; 259 Adiyaman University, Adiyaman, Turkey; 260 Bozok Universitetesi Rektörlügü, Yozgat, Turkey; 261 Marmara University, Istanbul, Turkey; 262 Milli Savunma University, Istanbul, Turkey; 263 Kafkas University, Kars, Turkey; 264 Istanbul Okan University, Istanbul, Turkey; 265 Hacettepe University, Ankara, Turkey; 266 Faculty of Engineering, Istanbul University-Cerrahpasa, Istanbul, Turkey; 267 Yildiz Technical University, Istanbul, Turkey; 268 Vrije Universiteit Brussel, Brussels, Belgium; 269 School of Physics and Astronomy, University of Southampton, Southampton, UK; 270 University of Bristol, Bristol, UK; 271 IPPP Durham University, Durham, UK; 272 Faculty of Science, Monash University, Clayton, Australia; 273 Università di Torino, Turin, Italy; 274 Bethel University, St. Paul, MN USA; 275 Karamanoğlu Mehmetbey University, Karaman, Turkey; 276 California Institute of Technology, Pasadena, CA USA; 277 United States Naval Academy, Annapolis, MD USA; 278 Bingol University, Bingol, Turkey; 279 Georgian Technical University, Tbilisi, Georgia; 280 Sinop University, Sinop, Turkey; 281 Erciyes University, Kayseri, Turkey; 282 Horia Hulubei National Institute of Physics and Nuclear Engineering (IFIN-HH), Bucharest, Romania; 283 Texas A &M University at Qatar, Doha, Qatar; 284 Kyungpook National University, Daegu, Korea; 285 Another Institute or International Laboratory Covered by a Cooperation Agreement with CERN, Geneva, Switzerland; 286 Universiteit Antwerpen, Antwerpen, Belgium; 287 Yerevan Physics Institute, Yerevan, Armenia; 288 Northeastern University, Boston, MA, USA; 289 Imperial College, London, UK; 290 Institute of Nuclear Physics of the Uzbekistan Academy of Sciences, Tashkent, Uzbekistan; 291 CERN, 1211 Geneva 23, Switzerland

## Abstract

A measurement is presented of a ratio observable that provides a measure of the azimuthal correlations among jets with large transverse momentum $p_{\textrm{T}}$. This observable is measured in multijet events over the range of $p_{\textrm{T}} = 360$–$3170\,\text {Ge}\hspace{-.08em}\text {V}$ based on data collected by the CMS experiment in proton-proton collisions at a centre-of-mass energy of 13$\,\text {Te}\hspace{-.08em}\text {V}$, corresponding to an integrated luminosity of 134$\,\text {fb}^{-1}$. The results are compared with predictions from Monte Carlo parton-shower event generator simulations, as well as with fixed-order perturbative quantum chromodynamics (pQCD) predictions at next-to-leading-order (NLO) accuracy obtained with different parton distribution functions (PDFs) and corrected for nonperturbative and electroweak effects. Data and theory agree within uncertainties. From the comparison of the measured observable with the pQCD prediction obtained with the NNPDF3.1 NLO PDFs, the strong coupling at the Z boson mass scale is $\alpha _\textrm{S} (m_{{\textrm{Z}}}) =0.1177 \pm 0.0013\, \text {(exp)} _{-0.0073}^{+0.0116} \,\text {(theo)} = 0.1177_{-0.0074}^{+0.0117}$, where the total uncertainty is dominated by the scale dependence of the fixed-order predictions. A test of the running of $\alpha _\textrm{S}$ in the $\,\text {Te}\hspace{-.08em}\text {V}$ region shows no deviation from the expected NLO pQCD behaviour.

## Introduction

In the standard model of particle physics, the strong interaction between partons (quarks and gluons) is described by the theory of quantum chromodynamics (QCD). A key property of the strong interaction is “asymptotic freedom”, which characterizes the decreasing value of the coupling $\alpha _\textrm{S} (Q)$ for increasingly larger momentum transfer *Q* that corresponds to smaller distances between the interacting partons. This property is a consequence of the non-Abelian nature of QCD, and can be theoretically derived from the renormalization group equations (RGE) [1–3]. Although the RGE cannot predict the absolute value of $\alpha _\textrm{S} (Q)$, they can accurately determine its evolution as a function of the energy scale *Q* [4]. By comparing experimental measurements to perturbative QCD (pQCD) predictions for a given observable, the value of $\alpha _\textrm{S} (Q)$ can be extracted at various scales [5, 6]. To compare various $\alpha _\textrm{S} (Q)$ determinations, it is standard practice to evolve them to a common scale given by the mass of the Z boson, $Q = m_{{\textrm{Z}}}$. The current world-average value of the QCD coupling at this reference scale is $\alpha _\textrm{S} (m_{{\textrm{Z}}}) = 0.1180 \pm 0.0009$ [5].

**Fig. 1:**
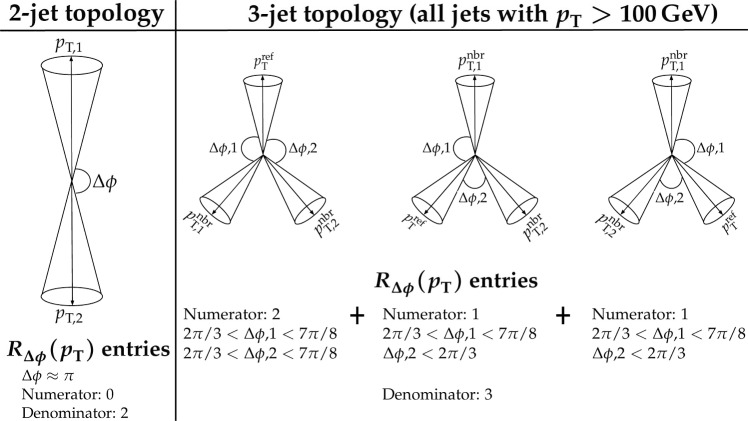
Example of the number of entries contributing to the numerator and denominator of the $R_{\varDelta \phi }(p_{\textrm{T}})$ ratio, Eq. (1), for 2-jet (left) and 3-jet (right) events, with all jets having $p_{\textrm{T}} > p_{\text {Tmin}}^{\text {nbr}} =100\,\text {Ge}\hspace{-.08em}\text {V}$. The 2-jet topology does not contribute (null numerator) to the $R_{\varDelta \phi }(p_{\textrm{T}})$ ratio when the azimuthal distance for neighbouring jets is fixed to $2\pi /3<\varDelta \phi <7\pi /8$. In the 3-jet topology, each jet is considered as a reference, and its azimuthal separations ($\varDelta \phi {,}1$ and $\varDelta \phi {,}2$) to other neighbouring jets (with $p_{\textrm{T,1}}^{\text{ nbr }}$ and $p_{\textrm{T,2}}^{\text{ nbr }}$) are computed. Each neighbouring jet with $\varDelta \phi$ within the specified interval increments the entries of the numerator, whereas the denominator simply counts the number of jets in the event

This paper reports a new extraction of the $\alpha _\textrm{S} (Q)$ coupling from multijet measurements at various energy scales in proton-proton (${\text {p}} {\text {p}}$) collisions at the CERN LHC. For this purpose, a ratio observable $R_{\varDelta \phi }(p_{\textrm{T}})$, related to the azimuthal correlations among jets, is measured as a function of the jet transverse momentum $p_{\textrm{T}}$. Similar ratio observables, based on either the distance in the plane of rapidity and azimuthal angle among jets, $R_{\varDelta R}(p_{\textrm{T}})$ [7], or on the dijet azimuthal decorrelations, $R_{\varDelta \phi }(H_\textrm{T})$ [8, 9], have already been used to extract the $\alpha _\textrm{S} (Q)$ coupling at hadron colliders. The $R_{\varDelta \phi }(p_{\textrm{T}})$ observable is defined as:

1 $$\begin{aligned} R_{\varDelta \phi }(p_{\textrm{T}}) = \frac{\sum _{i=1}^{N_{\text {jet}}(p_{\textrm{T}})} N_{\text {nbr}}^{(i)}(\varDelta \phi ,p_{\text {Tmin}}^{\text {nbr}})}{N_{\text {jet}}(p_{\textrm{T}})}, \end{aligned}$$

where the denominator $N_{\text {jet}}(p_{\textrm{T}})$ simply counts the number of jets in a given jet $p_{\textrm{T}}$ bin, and the numerator sums the number of neighbouring jets, $N_{\text {nbr}}^{(i)}$, around each jet *i* in the same $p_{\textrm{T}}$ bin. A neighbouring jet must exceed a minimum transverse momentum of $p_{\text {Tmin}}^{\text {nbr}}$ and be separated from jet *i* within a specified interval of azimuthal distance $\varDelta \phi$: ${\varDelta \phi }_{\text {min}}<\varDelta \phi <{\varDelta \phi }_{\text {max}}$. In fixed-order predictions of jet production based on pQCD calculations, the leading-order (LO) $2\rightarrow 2$ process is characterized by an azimuthal separation of $\varDelta \phi = \pi$. Since the sum in the numerator runs over all jets, this would lead to two entries at $p_{\textrm{T}} = p_\textrm{T,1} = p_\textrm{T,2}$. At next-to-leading order (NLO), the radiation of a third hard parton can give rise to a 3-jet topology with $\varDelta \phi$ between $2\pi /3$ and $\pi$ with respect to the jet opposite to the hemisphere with radiation (Fig. 1, right diagrams). Hence, by fixing the azimuthal distance for neighbouring jets to $2\pi /3<\varDelta \phi <7\pi /8$ in Eq. (1), the dijet case is avoided, and the numerator is different from zero only for events with three jets or more, whose LO cross section is proportional to $\alpha _\textrm{S} ^{3}$. Also here, a jet pair fulfilling both the selection in $p_{\text {Tmin}}^{\text {nbr}}$ and in $\varDelta \phi$ leads to two entries, but potentially at different jet $p_{\textrm{T}}$ values. On the other hand, the denominator corresponds to the inclusive jet cross section, which at LO is proportional to $\alpha _\textrm{S} ^{2}$, such that the $R_{\varDelta \phi }(p_{\textrm{T}})$ observable is directly proportional to $\alpha _\textrm{S}$, at the lowest order. A representative illustration, indicating the entries to the numerator and denominator of the $R_{\varDelta \phi }(p_{\textrm{T}})$ ratio, is shown in the left and right panels of Fig. 1 for a 2-jet and a 3-jet event, respectively.

In the ratio defined by Eq. (1) many experimental systematic uncertainties – such as those from the integrated luminosity, the jet energy scale (JES), and the jet energy resolution (JER) – cancel entirely or to a large extent. In addition, theoretical uncertainties – such as nonperturbative (NP) and parton distribution function (PDF) uncertainties – are reduced.

To rigorously account for correlations between the numerator and denominator, it is useful to consider the more general, two-dimensional jet-counting quantity, $N(p_{\textrm{T}},n)$, which is a function of the *i*-th jet’s $p_{\textrm{T}}$ and of the number *n* of neighbouring jets that satisfy the additional selection criteria for $p_{\text {Tmin}}^{\text {nbr}}$ and $\varDelta \phi$. Then, using $N(p_{\textrm{T}},n)$, it can be shown that the $R_{\varDelta \phi }(p_{\textrm{T}})$ observable can be also formulated as:

2 $$\begin{aligned} R_{\varDelta \phi }(p_{\textrm{T}}) = \frac{\sum _{n}nN(p_{\textrm{T}},n)}{\sum _{n}N(p_{\textrm{T}},n)}. \end{aligned}$$

Such a definition allows a multidimensional unfolding of the more general quantity $N(p_{\textrm{T}},n)$ to be performed, instead of a separate unfolding of the numerator and denominator of Eq. (1).

The measurement is performed using data collected with the CMS detector, during the LHC Run 2 data-taking period (2016–2018), corresponding to an integrated luminosity of 134$\,\text {fb}^{-1}$ at a centre-of-mass energy of 13$\,\text {Te}\hspace{-.08em}\text {V}$ [10–12]. Previous determinations of the strong coupling constant $\alpha _\textrm{S} (m_{{\textrm{Z}}})$ using jets at hadron colliders have been reported by the CDF [13] and D0 [7, 14] Collaborations in proton-antiproton collisions at $\sqrt{s}=1.8$ and 1.96$\,\text {Te}\hspace{-.08em}\text {V}$ at the Fermilab Tevatron. At the LHC, determinations have been reported using ${\text {p}} {\text {p}}$ collision data from the ATLAS and CMS Collaborations at $\sqrt{s}=7$ [15–22], 8 [9, 21–25], and 13 [26–29, 29–32]$\,\text {Te}\hspace{-.08em}\text {V}$.

The paper is organized as follows. In Sect. 2 a brief description of the CMS detector is given. In Sect. 3 the event reconstruction is described. Section 4 details the measurement of the $R_{\varDelta \phi }(p_{\textrm{T}})$ observable. Experimental results and theoretical predictions for the $R_{\varDelta \phi }(p_{\textrm{T}})$ observable are compared in Sect. 5. The determination of $\alpha _\textrm{S} (m_{{\textrm{Z}}})$ and the investigation of the running of the $\alpha _\textrm{S} (Q)$ coupling are presented in Sect. 6. Finally, a summary of the paper is given in Sect. 7.

Tabulated results are provided in the HEPData record for this analysis [33].

## The CMS detector

The central feature of the CMS apparatus is a superconducting solenoid of 6$\text {\,m}$ internal diameter, providing a magnetic field of 3.8$\text {\,T}$. Within the solenoid volume are a silicon pixel and strip tracker, a lead tungstate crystal electromagnetic calorimeter (ECAL), and a brass and scintillator hadron calorimeter (HCAL), each composed of a barrel and two endcap sections. Forward calorimeters extend the pseudorapidity coverage provided by the barrel and endcap detectors. Muons are measured in gas-ionization detectors embedded in the steel flux-return yoke outside the solenoid.

The electromagnetic calorimeter consists of 75 848 lead tungstate crystals, which provide coverage in pseudorapidity $|\eta | < 1.48$ in a barrel region (EB) and $1.48< |\eta | < 3.0$ in two endcap regions (EE). Preshower detectors consisting of two planes of silicon sensors interleaved with a total of three radiation lengths of lead are located in front of each EE detector.

In the region $|\eta | < 1.74$, the HCAL cells have widths of 0.087 in pseudorapidity and 0.087 in azimuth ($\phi$). In the $\eta$–$\phi$ plane, and for $|\eta | < 1.48$, the HCAL cells map on to $5{\times }5$ arrays of ECAL crystals to form calorimeter towers projecting radially outwards from close to the nominal interaction point. For $|\eta | > 1.74$, the coverage of the towers increases progressively to a maximum of 0.174 in $\varDelta \eta$ and $\varDelta \phi$. A more detailed description of the CMS detector, together with a definition of the coordinate system used and the relevant kinematic variables, can be found in Ref. [34].

Events of interest are selected using a two-tiered trigger system. The first level (L1), composed of custom hardware processors, uses information from the calorimeters and muon detectors to select events at a rate of around 100$\text {\,kHz}$ within a fixed latency of about 4$\,\upmu \text {s}$ [35]. The second level, known as the high-level trigger (HLT), consists of a farm of processors running a version of the full event reconstruction software optimized for fast processing and reduces the event rate to around 1$\text {\,kHz}$ before data storage [36].

## Event reconstruction

The global event reconstruction – also called particle-flow (PF) event reconstruction [37] – aims to reconstruct and identify each individual particle in an event, with an optimized combination of all subdetector information. In this process, the identification of the particle type (photon, electron, muon, charged hadron, neutral hadron) plays an important role in the determination of the particle direction and energy. Photons (e.g., coming from ${{{\varvec{\uppi }}}}^{0}$decays or from electron bremsstrahlung) are identified as ECAL energy clusters not linked to the extrapolation of any charged particle trajectory to the ECAL. Electrons (e.g., coming from photon conversions in the tracker material or from bottom quark (${\text {b }}$) hadron semileptonic decays) are identified as a primary charged particle track and potentially many ECAL energy clusters corresponding to this track extrapolation to the ECAL and to possible bremsstrahlung photons emitted along the way through the tracker material. Muons (e.g., from ${\text {b }}$hadron semileptonic decays) are identified as tracks in the central tracker consistent with either a track or several hits in the muon system, and associated with calorimeter deposits compatible with the muon hypothesis. Charged hadrons are identified as charged particle tracks neither identified as electrons, nor as muons. Finally, neutral hadrons are identified as HCAL energy clusters not linked to any charged-hadron trajectory, or as a combined ECAL and HCAL energy excess with respect to the expected charged-hadron energy deposit.

The energy of photons is obtained from the ECAL measurement. The energy of electrons is determined from a combination of the track momentum at the main interaction vertex, the corresponding ECAL cluster energy, and the energy sum of all bremsstrahlung photons attached to the track. The energy of muons is obtained from the corresponding track momentum. The energy of charged hadrons is determined from a combination of the track momentum and the corresponding ECAL and HCAL energies, corrected for the response function of the calorimeters to hadronic showers. Finally, the energy of neutral hadrons is obtained from the corresponding corrected ECAL and HCAL energies.

**Table 1:** The different HLT $p_{\textrm{T}}$ thresholds used in the measurement and the corresponding integrated luminosities for each data-taking year

$p_{\textrm{T}} ^{\text {thresh}}$ ($\text {Ge}\hspace{-.08em}\text {V}$)		40	60	80	140	200	260	320	400	450	500
	2016	0.0497	0.328	1.00	10.1	85.8	518	1526	4590	33,500	–
${\mathcal {L}}$ ($\text {pb}^{-1}$)	2017	0.182	0.505	2.53	26.6	189	469	1230	7690	9660	41,500
	2018	0.0151	0.419	2.17	47.1	202	466	1240	3720	7390	59,800

The primary vertex (PV) is taken to be the vertex corresponding to the hardest scattering in the event, evaluated using tracking information alone, as described in Section 9.4.1 of Ref. [38]. For each event, hadronic jets are clustered from the reconstructed particle candidates using the infrared and collinear safe anti-$k_{\textrm{T}}$ algorithm [39, 40] with a distance parameter of $R=0.7$. This choice of the parameter *R* enables the compatibility with previous results from the CMS Collaboration at $\sqrt{s}=7$ [16] and 13 [30]$\,\text {Te}\hspace{-.08em}\text {V}$. Jet momentum is determined as the vectorial sum of all particle momenta in the jet, and is found from simulation to be, on average, within 5 to 10% of the true momentum over the whole $p_{\textrm{T}}$ spectrum and detector acceptance. Additional ${\text {p}} {\text {p}}$ interactions within the same, or nearby, bunch crossings (pileup) can contribute additional tracks and calorimetric energy depositions to the jet momentum. To mitigate this effect, charged particles identified to be originating from pileup vertices are discarded, and an offset correction is applied to correct for the remaining contributions [41]. The JES corrections are derived from simulation to bring the measured response of jets to that of particle-level jets on average. In situ measurements of the momentum balance in dijet, $\text {photon} + \text {jet}$, ${\textrm{Z}} + \text {jet}$, and multijet events are used to account for any residual differences in the jet energy scale between the measured data and simulation [42]. The jet energy resolution amounts typically to 15–20% at 30$\,\text {Ge}\hspace{-.08em}\text {V}$, 10% at 100$\,\text {Ge}\hspace{-.08em}\text {V}$, and 5% at 1$\,\text {Te}\hspace{-.08em}\text {V}$ [42]. Additional selection criteria are applied to each jet to remove jets potentially dominated by anomalous contributions from various subdetector components or reconstruction failures [43].

The missing transverse momentum vector ${\vec p}_{\textrm{T}}^{\hspace{1.66656pt}\text {miss}}$ is computed as the negative vector sum of the transverse momenta of all the PF candidates in an event, and its magnitude is denoted as $p_{\textrm{T}} ^\text {miss}$ [44]. The ${\vec p}_{\textrm{T}}^{\hspace{1.66656pt}\text {miss}}$ is modified to account for corrections to the energy scale of the reconstructed jets in the event.

During the 2016-2017 data taking, a gradual shift in the timing of the inputs of the ECAL L1 trigger in the region at $|\eta | > 2.0$ caused a specific trigger inefficiency [45], called “prefiring” hereafter. For events containing a jet with $p_{\textrm{T}} \gtrsim 100\,\text {Ge}\hspace{-.08em}\text {V}$ in the region $2.5< |\eta | < 3.0$, the efficiency loss is $\approx$10–20%, depending on $p_{\textrm{T}}$, $\eta$, and data-taking period.

## Data analysis

### Event selection criteria

Each event is required to have at least one offline-reconstructed PV with *z* coordinate satisfying the criterion $|z_\textrm{PV} | <24\,\text {cm}$ and radial distance from the interaction point $\rho _{\textrm{PV}}<2\,\text {cm}$. Anomalous high-$p_{\textrm{T}} ^\text {miss}$ events can be due to a variety of reconstruction failures, detector malfunctions, or noncollision backgrounds. Such events are rejected by event filters that are designed to identify more than 85–90% of the spurious high-$p_{\textrm{T}} ^\text {miss}$ events with a mistagging rate less than 0.1% [44]. Only events that have been accepted by at least one single-jet trigger path (described in Sect. 4.2) are included in the measurement. For the rejection of poorly reconstructed jets and jets originating from detector noise, additional quality criteria are applied to them based on their constituents [43].

The measurement is based on an inclusive jet sample that contains only jets reconstructed within the rapidity range $|y | < 2.5$ and with transverse momenta $p_{\textrm{T}} >50\,\text {Ge}\hspace{-.08em}\text {V}$. The $p_{\text {Tmin}}^{\text {nbr}}$ threshold and azimuthal separation interval for neighbouring jets as defined in Eq. (1), are set to 100$\,\text {Ge}\hspace{-.08em}\text {V}$ and $2\pi /3<\varDelta \phi <7\pi /8$, respectively. This choice was motivated by statistical optimization, extending the phase space as much as possible, and guaranteeing that the NLO predictions remain valid and soft effects are negligible.

### Triggers

The analysis is based on single-jet triggers that require at least one jet with $p_{\textrm{T}}$ above a given threshold $(p_{\textrm{T}} ^{\text {thresh}})$ to be present in the event. Table 1 shows the different HLT thresholds along with the effective integrated luminosities recorded by the triggers for 2016, 2017 and 2018. All triggers in Table 1 were prescaled, apart from trigger 450 for 2016, and trigger 500 for 2017 and 2018. The efficiency for each trigger is estimated as a function of leading-jet $p_{\textrm{T}}$ using a lower-threshold trigger, except for the lowest-threshold trigger efficiency. The latter is estimated using a tag-and-probe method applied to dijet topologies, which counts the jets reconstructed with the offline PF algorithm that can be matched to HLT jets, considering only the jets leading and subleading in $p_{\textrm{T}}$. The data consist of events selected with a combination of triggers in mutually exclusive leading-jet $p_{\textrm{T}}$ intervals. The usage of a specific trigger is enabled only in phase-space regions where its efficiency is larger than 99.5% and disabled in phase-space regions where the efficiency of a higher-threshold trigger is larger than 99.5%. The jet-counting variables are combined event-by-event by applying weights to account for the trigger prescales of each data sample.

**Table 2:** Values of the $R_{\varDelta \phi }(p_{\textrm{T}})$ observable in different $p_{\textrm{T}}$ intervals, and associated experimental uncertainties

$p_{\textrm{T}}$ ($\text {Ge}\hspace{-.08em}\text {V}$)	$R_{\varDelta \phi }(p_{\textrm{T}})$	Stat. (%)	JES (%)	Prefiring (%)	JER (%)	PU (%)	$\text {MC}_{\text {model}}$ (%)
360–430	0.25	0.26	0.74	0.06	0.08	0.01	–
430–510	0.26	0.23	0.69	0.09	0.06	0.02	–
510–600	0.27	0.19	0.67	0.12	0.05	0.02	–
600–700	0.27	0.18	0.66	0.13	0.04	0.02	0.01
700–800	0.28	0.18	0.65	0.12	0.04	0.02	0.01
800–920	0.28	0.20	0.65	0.10	0.04	0.02	0.01
920–1050	0.27	0.27	0.66	0.07	0.05	0.02	–
1050–1190	0.27	0.39	0.67	0.05	0.06	0.02	–
1190–1340	0.27	0.58	0.70	0.03	0.07	0.02	–
1340–1500	0.26	0.90	0.75	0.02	0.08	0.02	–
1500–1680	0.26	1.34	0.84	0.01	0.11	0.02	–
1680–1870	0.25	2.16	0.98	–	0.14	0.02	–
1870–2070	0.24	3.42	1.21	–	0.19	0.02	–
2070–2300	0.21	5.66	1.62	–	0.27	0.02	–
2300–2560	0.22	8.23	2.36	–	0.39	0.02	–
2560–3170	0.21	10.49	5.00	–	0.77	0.01	–

### Unfolding

To compare the experimental data with theoretical predictions, the measured distributions must be corrected for detector effects, such as finite $p_{\textrm{T}}$ resolution and limited detector acceptance. The detector effects are parameterized through a response matrix built from simulated event samples using pythia  8.240 [46] with tunes CUETP8M1 [47] and CP5 [48], where reconstructed-level jets are matched to generator-level jets as explained next. First, the generated jets in each event are ordered by decreasing $p_{\textrm{T}}$. Then, each generated jet is matched to the reconstructed jet with the highest $p_{\textrm{T}}$, within a cone of radius $\varDelta R= \sqrt{\smash [b]{(\varDelta \eta )^2+(\varDelta \phi )^2}} = 0.35$ (where $\varDelta \eta$ and $\varDelta \phi$ are the angular differences in pseudorapidity and azimuthal angle between the generated and reconstructed jets). The probability matrix ${\varvec{A}}$ corresponds to the row-by-row normalized response matrix. Each element $A_{ij}$ represents the probability of a jet produced in (generator-level) bin *j* to be observed in (reconstructed-level) bin *i*. The detector effects are corrected through an unfolding procedure that accounts for bin migrations, background (fake jets, i.e., reconstructed-level jets that could not be matched to generator-level jets), and inefficiencies (missed jets, i.e., generator-level jets that could not be matched to reconstructed-level jets), and corrects the measurement from the detector level to the level of stable particles (except neutrinos) with mean decay-lengths larger than $c\tau =10\text {\,mm}$ (where $\tau$ denotes the mean proper lifetime of the particle).

The unfolding procedure is implemented using the TUnfold package [49]. The determination of the particle-level distribution (${\varvec{x}}$) is performed with the matrix pseudoinverse method [50] using a detector-level distribution (${\varvec{y}}$) with twice the number of bins of the particle-level distribution. The latter are defined in Table 2 (first column) and are chosen to ensure that the bin sizes remain at least twice as large as the jet $p_{\textrm{T}}$ resolution. The unfolding solution arises from the minimization of the quantity

3 $$\begin{aligned} \chi ^2 = {\varvec{\left( Ax + b - y\right) ^T (V^{-1}) \left( Ax + b - y\right) }}, \end{aligned}$$

where ${\varvec{b}}$ is the background obtained from simulated events, and ${\varvec{V}}$ is the covariance matrix (corrected for the background) of the detector-level data including their statistical uncertainties and correlations. Instead of unfolding separately the numerator and denominator of Eq. (1), a multidimensional unfolding of the more general, equivalent quantity $N(p_{\textrm{T}},n)$ is performed, which rigorously accounts for the numerator-denominator statistical correlations following Eq. (2).

**Fig. 2:**
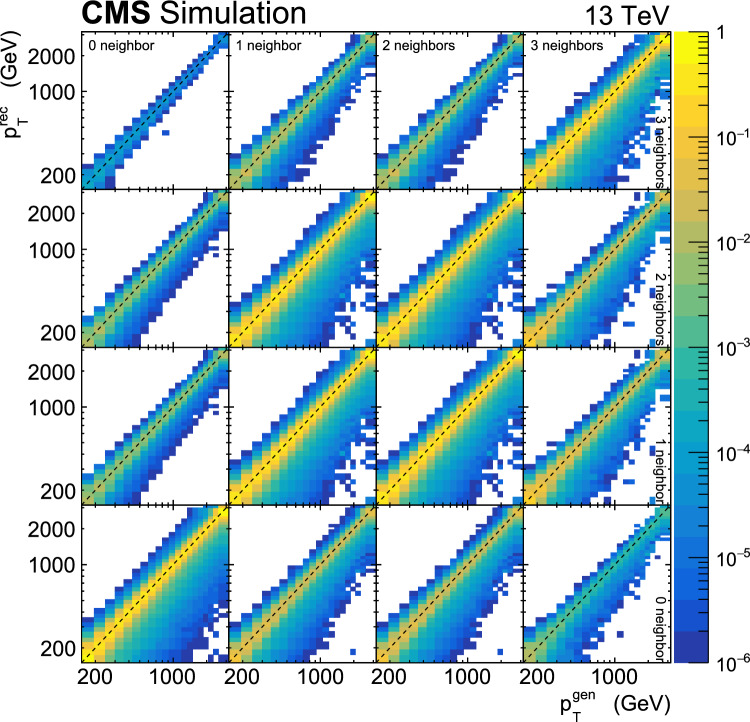
Probability matrix for the $N(p_{\textrm{T}},n)$ distribution built using pythia8 simulated events. The horizontal axis corresponds to the generator-level jet $p_{\textrm{T}}$, and the vertical axis to the reconstructed-level jet $p_{\textrm{T}}$. The $4\times 4$ structure of the matrix corresponds to the bins of neighbouring jets *n* (labelled in the uppermost row and rightmost column), and indicates migrations among those bins. The horizontal and vertical axes of each cell correspond to the $p_{\textrm{T}}$ of the jets, and each cell indicates the migrations among the jet $p_{\textrm{T}}$ bins. The range of colours covers from $10^{-6}$ to 1, and indicates the probability of migrations from a given (generator) particle-level bin to the corresponding (reconstructed) detector-level bin

Figure 2 shows the probability matrix for the $N(p_{\textrm{T}},n)$ quantity. The number of events with $n\ge 4$ is small, and $n=3$ is the maximum number of neighbouring jets shown here. The condition number (defined as the absolute value of the ratio between the largest and smallest matrix eigenvalues) for the matrix ${\varvec{A}}$ is ${\approx }5.5$, which means that the unfolding problem is well-conditioned, and therefore no additional regularization is required.

### Experimental uncertainties

**Fig. 3:**
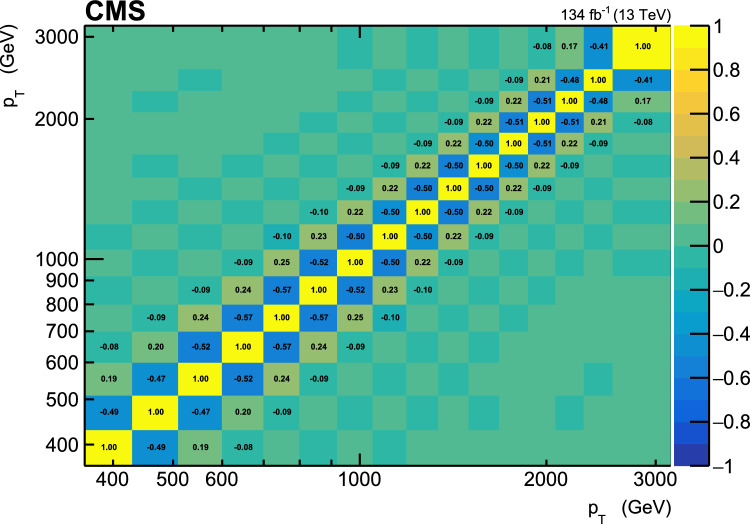
Bin-to-bin correlation matrix for the $R_{\varDelta \phi }(p_{\textrm{T}})$ distribution at the particle level, where the value 1 $(-1)$ corresponds to fully (anti)correlated bins. For illustration purposes, only bins with (anti)correlations larger (smaller) than 0.05 $(-0.05)$ are shown also as text

The experimental uncertainties contain statistical and systematic sources that propagate to the measured distributions. The statistical uncertainties are obtained from the covariance matrix, extracted at the particle level from the $N(p_{\textrm{T}},n)$ distribution along with the $R_{\varDelta \phi }(p_{\textrm{T}})$ observable, as described in Sect. 4.3. The bin-to-bin correlation matrix at the particle level is shown in Fig. 3, where the value 1 $(-1)$ corresponds to fully (anti)correlated bins. The diagonal elements of the correlation matrix are by construction always unity, and the off-diagonal elements represent the bin-to-bin (anti)correlations, where the highest (lowest) value is 0.49 ($-0.57$). The statistical uncertainties in the $R_{\varDelta \phi }(p_{\textrm{T}})$ measurement remain below 1% up to ${\approx }1.5\,\text {Te}\hspace{-.08em}\text {V}$ increasing to about 10% at ${\approx }3\,\text {Te}\hspace{-.08em}\text {V}$.

The calibration of the reconstructed jet energy is performed through a series of successive stages implemented in the JES correction procedure [42], as described in Sect. 3. The JES uncertainty is composed of 27 individual uncorrelated contributions, which are investigated one-by-one considering a $\pm 1$ standard-deviation variation from their nominal value. Each variation is applied at the detector level and propagated to the particle-level measurement by repeating the unfolding procedure. Finally, the total JES uncertainty is computed as the quadratic sum of individual JES uncertainty sources, and remains below 1% up to ${\approx }1.5\,\text {Te}\hspace{-.08em}\text {V}$ increasing to about 5% at ${\approx }3\,\text {Te}\hspace{-.08em}\text {V}$. Additional variations of the trigger prefiring corrections described in Sect. 3 are applied in the same manner. The uncertainties from prefiring corrections in the $R_{\varDelta \phi }(p_{\textrm{T}})$ measurement are smaller than 0.13%.

In simulated samples, a detailed modelling of the CMS detector is included based on the Geant4 toolkit [51]. The JER obtained in the detector simulation is generally better than that in the actual detector. Therefore, the energy of reconstructed jets in simulation is smeared out, so that the simulated JER matches the one measured in experimental data. The JER uncertainty is estimated by varying the smearing factors within $\pm 1$ standard deviations from their nominal values, and propagated to the particle-level measurement by repeating the unfolding procedure. The JER uncertainty in the $R_{\varDelta \phi }(p_{\textrm{T}})$ measurement is below 0.8%.

The probability distribution for the number of ${\text {p}} {\text {p}}$ interactions per bunch crossing is represented by pileup (PU) profiles. To account for differences between the measured and simulated PU profiles, the simulated events are reweighted using the PU distribution of the experimental data as a reference. An additional systematic uncertainty, which remains below 0.03%, is evaluated by varying the PU profile correction in the simulation. The model dependence introduced by unfolding is estimated from the difference in the $R_{\varDelta \phi }(p_{\textrm{T}})$ distribution unfolded with response matrices obtained from pythia8 and MadGraph 5_amc@nlo  2.6 [52, 53] interfaced with pythia8. Additionally, the model dependence for inefficiencies (missed jets) and backgrounds (misreconstructed jets) is studied by varying separately their rates within an estimate of 5%, which largely covers the model dependence of migrations in and out of the phase space. The total model dependence uncertainties ($\text {MC}_{\text {model}}$) are negligible (${<}0.01\%$) compared with other uncertainties for the bulk of the spectrum. The trigger efficiency uncertainties are also negligible in the $R_{\varDelta \phi }(p_{\textrm{T}})$ measurement. The $R_{\varDelta \phi }(p_{\textrm{T}})$ observable values along with all the experimental uncertainties are shown in Table 2.

**Fig. 4:**
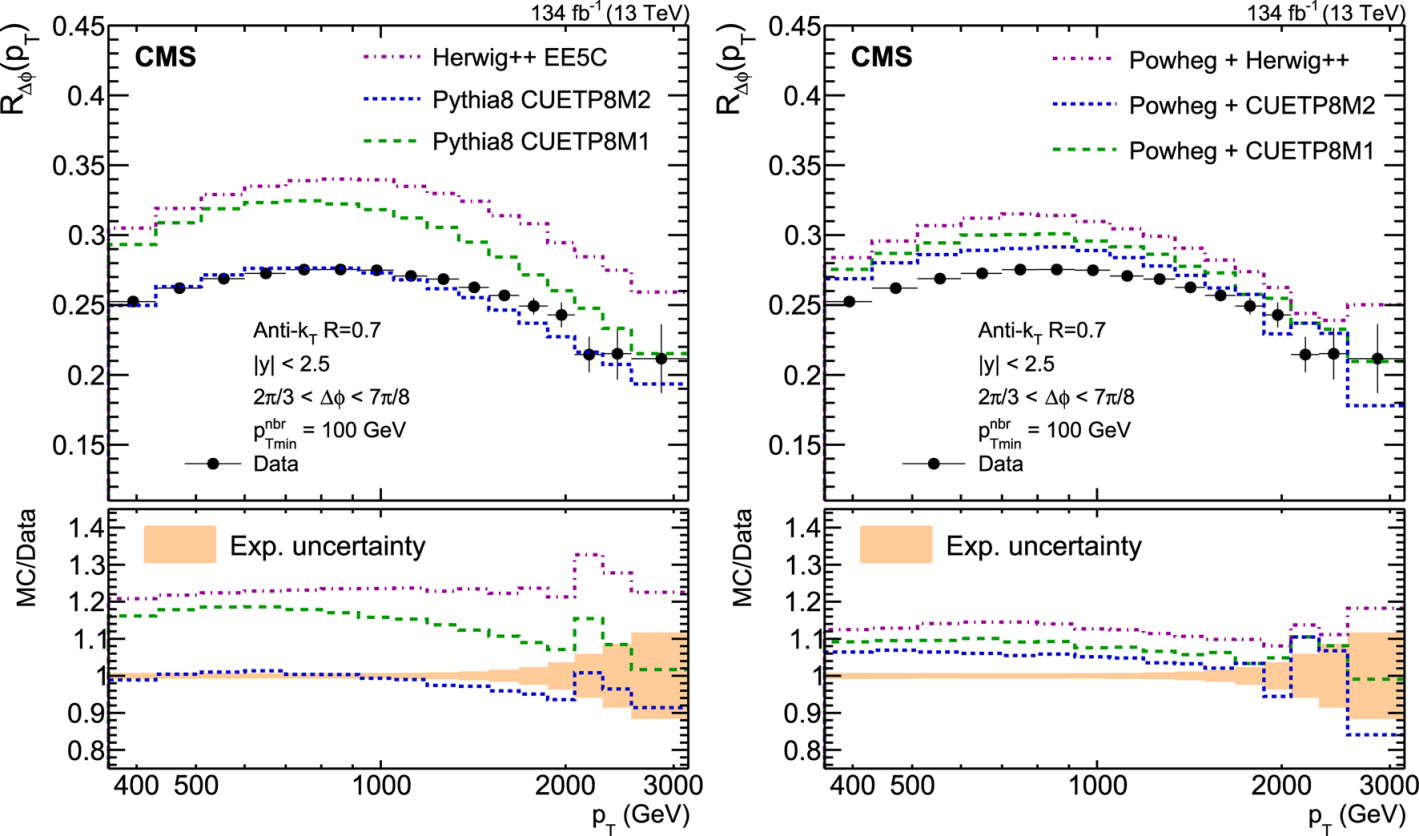
The $R_{\varDelta \phi }(p_{\textrm{T}})$ observable as a function of $p_{\textrm{T}}$, compared with MC generator predictions at LO (left) and at NLO (right) accuracy. The LO predictions are obtained with pythia8 tunes CUETP8M1 and CUETP8M2, and herwig++ tune UE-EE-5-CTEQ6L1 MC event generators. The NLO predictions are obtained with powheg interfaced with each of the aforementioned MC event generators. The experimental data are represented with black dots and the MC predictions with coloured lines. The lower panel of each plot shows the ratio between MC predictions and experimental data. The total experimental uncertainties are indicated by the vertical error bars (upper panels) and coloured band (lower panels) correspondingly

**Table 3:** Default and range of $\alpha _\textrm{S} (m_{{\textrm{Z}}})$ values used in the different NLO PDF sets

PDF set	Default $\alpha _\textrm{S} (m_{{\textrm{Z}}})$	Alternative $\alpha _\textrm{S} (m_{{\textrm{Z}}})$
ABMP16 [78]	0.1191	0.114–0.123
CT18 [79]	0.1180	0.110–0.124
MSHT20 [80]	0.1200	0.108–0.130
NNPDF3.1 [81]	0.1180	0.106–0.130

**Fig. 5:**
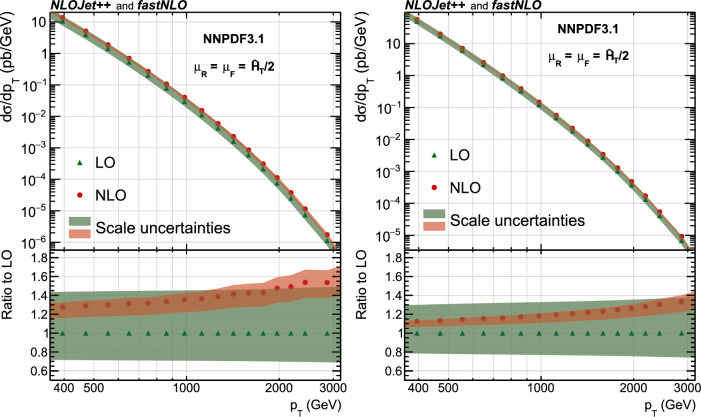
Theoretical predictions for the cross sections corresponding to the numerator (left) and denominator (right) of the $R_{\varDelta \phi }(p_{\textrm{T}})$ ratio, Eq. (1), obtained using the NNPDF3.1 NLO PDF set. The coloured bands represent the LO and NLO scale uncertainties derived with a six-point variation of $\mu _\textrm{R}$ and $\mu _\textrm{F}$ from the central reference value. The lower panels show the ratios to the respective LO predictions

**Fig. 6:**
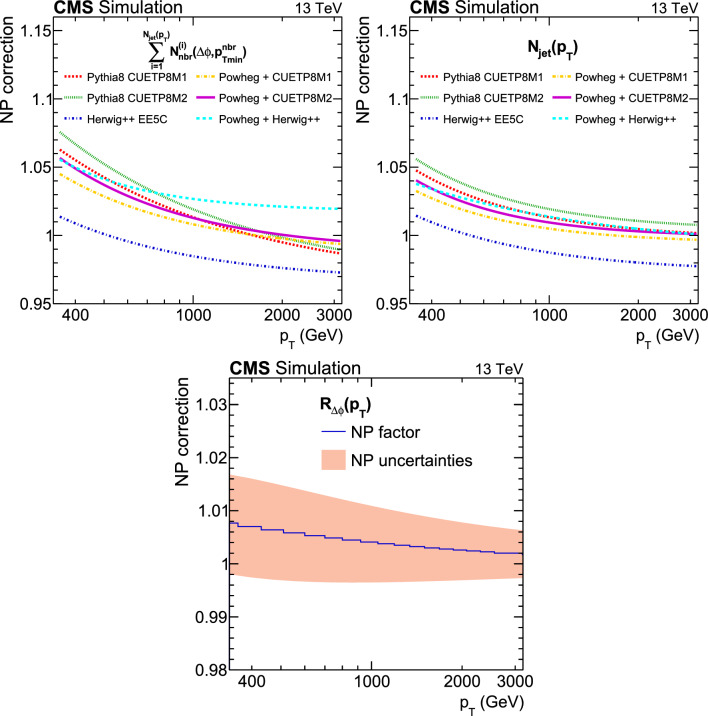
Nonperturbative correction factors for the numerator (upper left) and denominator (upper right) of the $R_{\varDelta \phi }(p_{\textrm{T}})$ ratio, Eq. (1), using pythia8 with tunes CUETP8M1 and CUETP8M2, herwig++ with tune UE-EE-5-CTEQ6L1, and POWHEG interfaced with each of them. The lower plot shows the NP correction factors (blue line) for $R_{\varDelta \phi }(p_{\textrm{T}})$ and their uncertainties

## Theoretical predictions

### Monte Carlo event generators predictions

Experimental data are compared with predictions from herwig++  2.7.1 [54], pythia  8.240 [46], and powheg  2.0 [55] Monte Carlo (MC) event generators obtained using the Rivet toolkit [56]. The herwig++ event generator computes the matrix elements (MEs) at LO accuracy for $2 \rightarrow 2$ QCD scattering processes. The parton shower (PS) is simulated through successive angular-ordered emissions, and the cluster fragmentation model [57] is used for the hadronization. The underlying event (UE) activity is obtained from the simulation of multiparton interactions (MPIs) tuned to experimental data. The set of herwig++ parameters used in this analysis is that of the UE-EE-5-CTEQ6L1 tune [58] based on the CTEQ6.1M LO PDF set [59]. Similarly to herwig++, in pythia8 the MEs are calculated at LO accuracy for $2 \rightarrow 2$ QCD scattering processes. The PS is simulated through successive $p_{\textrm{T}}$-ordered emissions and the hadronization mechanism employs the Lund string model [60]. Two different sets of parameters are used for pythia8, the CUETP8M1 tune [47] based on the NNPDF2.3 LO PDF set [61, 62] and the CUETP8M2 tune [63] based on the NNPDF3.0 LO PDF set [64]. The powheg [65, 66] generator, based on the Powheg box [55], generates $2 \rightarrow 2$ matrix elements at NLO accuracy, as well as $2 \rightarrow 3$ matrix elements at LO accuracy and uses the NNPDF3.0 NLO PDF set [64]. To simulate the PS, hadronization, and MPI processes, powheg is interfaced either with pythia8 or with herwig++.

Figure 4 (left) shows the particle-level data for the $R_{\varDelta \phi }(p_{\textrm{T}})$ observable compared with the predictions of pythia8 tunes CUETP8M1 and CUETP8M2, and herwig++ tune UE-EE-5-CTEQ6L1 LO MC event generators. The lower panels show the corresponding ratios between the MC predictions and the measured data. Figure 4 (right) illustrates the particle-level $R_{\varDelta \phi }(p_{\textrm{T}})$ observable compared with powheg interfaced with pythia8 tunes CUETP8M1 and CUETP8M2, and herwig++ tune UE-EE-5-CTEQ6L1 results. The corresponding lower panels show the ratios between the powheg predictions and the measurement. The coloured band on both lower panels represents the total experimental uncertainties.

From 360 up to around 800$\,\text {Ge}\hspace{-.08em}\text {V}$, the $R_{\varDelta \phi }(p_{\textrm{T}})$ distribution rises as the phase space for the production of a third jet increases. Then, the distribution reaches a plateau up to around 1200$\,\text {Ge}\hspace{-.08em}\text {V}$, followed by a decrease due to the running of $\alpha _\textrm{S}$, and the reduced amount of gluon scatterings.

The predictions from LO herwig++ and LO pythia8 tune CUETP8M1 overestimate the measurement by ${\approx }20\%$ and ${\approx }12$–18%, respectively. On the other hand, the predictions from the (LO) pythia8 tune CUETP8M2 give a good description of the data. Besides the PDF set, the main differences between the parameters of the two pythia8 tunes are the value of $\alpha _\textrm{S}$ used for the initial-state shower $\alpha _\textrm{S} ^{\text {ISR}}$, the MPI infrared regularization scale $p_{\text {T0}}^{\text {ref}}$, and the amount of colour reconnection. Among the NLO MC predictions based on powheg, the powheg interfaced with pythia8 tune CUETP8M2 gives the best description, being ${\approx }5$–6% away from the measurement. Finally, powheg interfaced with herwig++ tune UE-EE-5-CTEQ6L1 or with pythia8 tune CUETP8M1 overestimate the $R_{\varDelta \phi }(p_{\textrm{T}})$ measurement by ${\approx } 12\%$ and ${\approx }10\%$, respectively.

### Fixed-order predictions

Fixed-order theoretical predictions for the $R_{\varDelta \phi }(p_{\textrm{T}})$ observable are obtained up to NLO accuracy in pQCD with the NLOJet++ program [67, 68] within the fastNLO framework [69, 70]. The predictions are extracted for several PDF sets available via the lhapdf library [71], using their default value for the strong coupling constant $\alpha _\textrm{S} (m_{{\textrm{Z}}})$, and alternative values, as shown in Table 3. The central reference values $\mu _0$ for the renormalization ($\mu _\textrm{R}$) and factorization ($\mu _\textrm{F}$) scales are defined as:

4 $$\begin{aligned} \mu _\textrm{R} =\mu _\textrm{F} =\hat{H}_{\textrm{T}}/2, \end{aligned}$$

where $\hat{H}_{\textrm{T}}$ is the scalar sum of the transverse momenta of all partons in the event. This choice follows recommendations detailed in Ref. [72], which favour $\hat{H}_{\textrm{T}}$ over $p_{\textrm{T},\text {jet}}$ and discourage the use of $p_{\textrm{T},\text {max}}=p_\textrm{T,1}$ as the central scale choice for inclusive jet cross sections. For 3-jet ratio observables such as $R_{\varDelta \phi }(p_{\textrm{T}})$, Refs. [73, 74] conclude that $\hat{H}_{\textrm{T}}/2$ is slightly preferred for comparisons with theoretical predictions at next-to-NLO accuracy. The uncertainties related to missing higher-order terms of the perturbative series are estimated using the conventional recipe [75–77], i.e., by varying $\mu _\textrm{R}$ and $\mu _\textrm{F}$ around the reference scale $\mu _{0}$ within six combinations: $(\mu _\textrm{R}/\mu _{0},\mu _\textrm{F}/\mu _{0})=(1/2,1/2)$, (1/2, 1), (1, 1/2), (1, 2), (2, 1), (2, 2). An envelope is constructed from the various combinations, where the edges define the scale uncertainties.

**Fig. 7:**
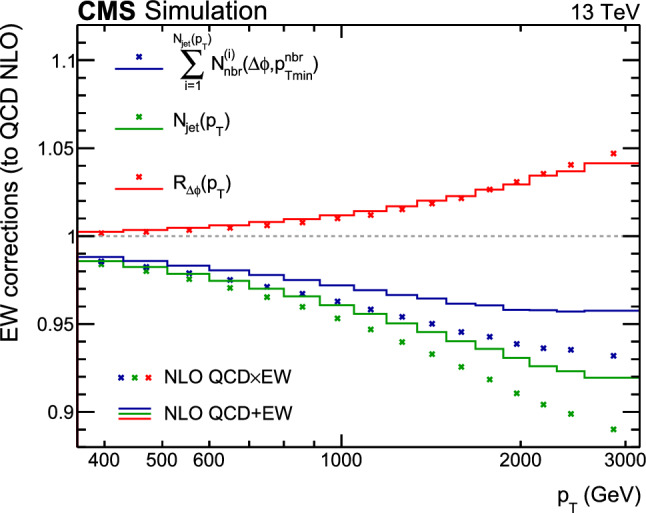
Electroweak corrections for the numerator (blue) and denominator (green) of Eq. (1), and for the $R_{\varDelta \phi }(p_{\textrm{T}})$ ratio itself (red). The solid lines correspond to the additive combination of NLO EW corrections to the QCD process (NLO QCD$\,+\,$EW), and the markers represent the multiplicative combination (NLO QCD$\,\times \,$EW)

**Fig. 8:**
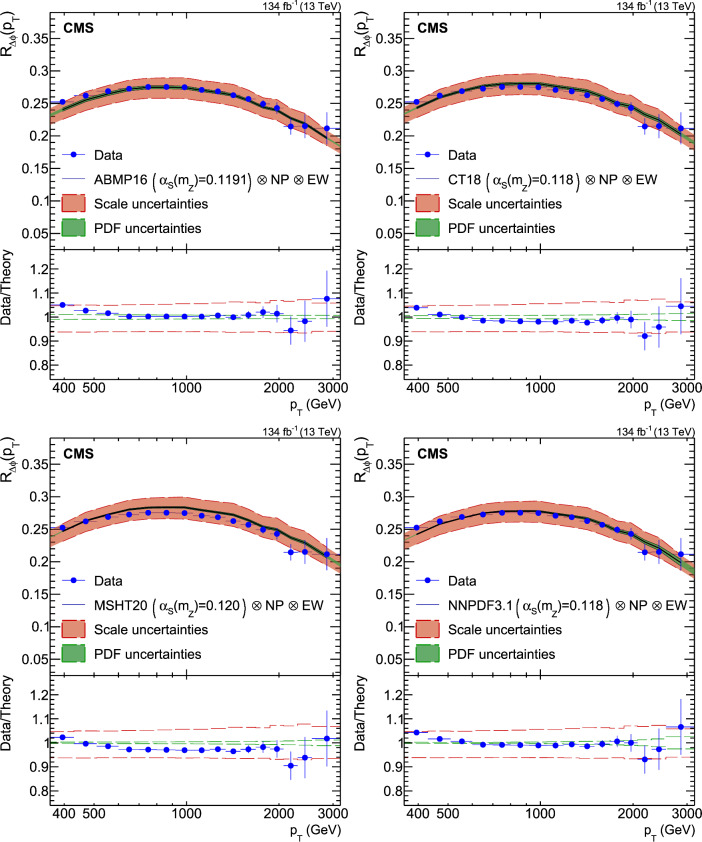
The $R_{\varDelta \phi }(p_{\textrm{T}})$ observable as a function of $p_{\textrm{T}}$, compared with fixed-order theoretical calculations at NLO accuracy using the ABMP16 (upper left), CT18 (upper right), MSHT20 (lower left), and NNPDF3.1 (lower right) NLO PDF sets. The experimental data are indicated with blue dots (with error bars representing the total experimental uncertainty), the theoretical prediction for the default $\alpha _\textrm{S} (m_{{\textrm{Z}}})$ for each PDF set with black solid lines, the scale uncertainties with red bands, and the PDF uncertainties with green bands. The lower panel of each plot shows the ratio between experimental data and theoretical predictions

Theoretical calculations are performed separately for the cross sections corresponding to the jet counts in the numerator and denominator of the $R_{\varDelta \phi }(p_{\textrm{T}})$ ratio defined in Eq. (1). The predictions using NNPDF3.1 for the numerator (left) and denominator (right) differential cross sections ($\textrm{d}\sigma /\textrm{d}p_{\textrm{T}}$) at LO and NLO accuracy are shown in Fig. 5, along with the scale uncertainties (coloured bands). The lower panels in this figure display the ratios to the respective LO predictions, where the so-called NLO pQCD *K* factors (NLO/LO) are about 1.30–1.55 for the numerator and 1.20–1.35 for the denominator (1.08–1.15 for their ratio). The LO and NLO scale uncertainty bands overlap over the whole phase space. The NLO scale uncertainties are in the range 9–17% for the numerator and 5–10% for the denominator.

To compare fixed-order predictions at parton level with unfolded data, the former must be corrected for NP effects due to MPI and hadronization (HAD). Based on PS generators, the NP correction factors are evaluated from the ratio of the nominal over the generated cross sections when MPI and HAD effects are switched off:

5 $$\begin{aligned} C^{\text {NP}}=\frac{\sigma ^{\text {PS+MPI+HAD}}}{\sigma ^{\text {PS}}}. \end{aligned}$$

The model dependence of $C^{\text {NP}}$ is investigated using different MC event generators, namely pythia8 with tunes CUETP8M1 and CUETP8M2, herwig++ with tune UE-EE-5-CTEQ6L1, and powheg interfaced with each one of them. A simple polynomial function $a + b p_{\textrm{T}} ^c$ (where *a*, *b*, and *c* are free parameters) is used to parameterize the dependence of $C^{\text {NP}}$ on jet $p_{\textrm{T}}$ for each MC event generator, to avoid statistical fluctuations in less populated regions of phase space. An envelope is constructed from the different MC predictions, where the central values are identified as the NP correction factors $C^{\text {NP}}$ and the edges define the corresponding uncertainties. Figure 6 shows the NP correction factors obtained for the numerator (upper left) and denominator (upper right) of the $R_{\varDelta \phi }(p_{\textrm{T}})$ observable. The lower panels show the final NP correction factors $C^{\text {NP}}$ (blue line) for $R_{\varDelta \phi }(p_{\textrm{T}})$. The red band is constructed from the envelope of individual ratios and represents the relevant uncertainties, which are less than 1%.

**Fig. 9:**
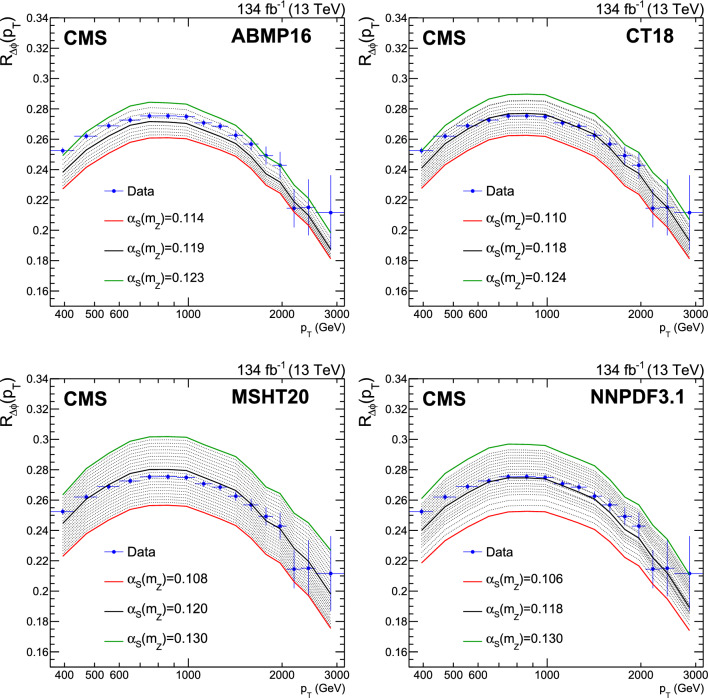
Sensitivity of the $R_{\varDelta \phi }(p_{\textrm{T}})$ ratio to the strong coupling constant $\alpha _\textrm{S} (m_{{\textrm{Z}}})$. The data are indicated with blue dots with error bars representing the total experimental uncertainty. In each plot, the lines represent fixed-order NLO theoretical calculations obtained with ABMP16 (upper left), CT18 (upper right), MSHT20 (lower left) and NNPDF3.1 (lower right) NLO PDF sets. Solid green (red) lines indicate maximum (minimum) values, and dotted black lines intermediate values of $\alpha _\textrm{S} (m_{{\textrm{Z}}})$ for each PDF set

To further improve the accuracy, in particular at large jet $p_{\textrm{T}}$, the theoretical predictions are complemented with electroweak (EW) corrections. The complete set of NLO corrections for three-jet production at the LHC is presented in Ref. [82]. To obtain the EW corrections for the $R_{\varDelta \phi }(p_{\textrm{T}})$ observable, the sherpa event generator [83] is used, interfaced with recola [84, 85]. Further details on the implementation of the above interface as well as on the method used for the subtraction of NLO EW infrared divergences are reported in Refs. [86, 87], respectively. The pure NLO EW corrections for *n*-jet production are defined as:

6 $$\begin{aligned} \sigma _{\textrm{nj}}^{\text {NLO EW}} = \sigma _{\textrm{nj}}^{\text {LO}} + \sigma _{\textrm{nj}}^{\varDelta \text {NLO}_1}, \end{aligned}$$

where $\sigma _{\textrm{nj}}^{\text {LO}}$ is the pure LO pQCD cross section and $\varDelta \text {NLO}_1$ accounts for the virtual and real EW corrections. The additive and multiplicative combination of the above corrections to the cross sections are defined, respectively, as:

7 $$\begin{aligned} \sigma _{\textrm{nj}}^{\text {NLO QCD+EW}}= &   \sigma _{\textrm{nj}}^{\text {LO}} + \sigma _{\textrm{nj}}^{\varDelta \text {NLO}_0} + \sigma _{\textrm{nj}}^{\varDelta \text {NLO}_1}, \end{aligned}$$

8 $$\begin{aligned} \sigma _{\textrm{nj}}^{\text {NLO QCD}\times \text {EW}}= &   \sigma _{\textrm{nj}}^{\text {LO}}\left( 1\!+\!\frac{\sigma _{\textrm{nj}}^{\varDelta \text {NLO}_0}}{\sigma _{\textrm{nj}}^{\text {LO}}}\right) \left( 1\!+\!\frac{\sigma _{\textrm{nj}}^{\varDelta \text {NLO}_1}}{\sigma _{\textrm{nj}}^{\text {LO}}}\right) , \end{aligned}$$

where $\varDelta \text {NLO}_0$ accounts for the virtual and real QCD corrections. Figure 7 shows the EW corrections obtained for the numerator and denominator cross sections, and for the $R_{\varDelta \phi }(p_{\textrm{T}})$ ratio. The multiplicative combination, Eq. (8), is considered as the main result, whereas the additive combination, Eq. (7) is used as an uncertainty estimate. The relative change of the central $\alpha _\textrm{S} (m_{{\textrm{Z}}})$ result (Sect. 6), when the additive combination is used as the main result, is smaller than 0.2%. The EW corrections for $R_{\varDelta \phi }(p_{\textrm{T}})$ observable range from 0.2 to 5.0% and their relevant uncertainties from 0.01 to 0.53%.

Comparisons between the measurement and the theoretical predictions for the four different PDF sets are shown in Fig. 8. The PDF uncertainties in the $R_{\varDelta \phi }(p_{\textrm{T}})$ predictions are evaluated at 68% confidence level for each PDF set following either the Hessian [88] or the MC [89] methods, and are about 1–2% in all cases. The scale uncertainties in $R_{\varDelta \phi }(p_{\textrm{T}})$ predictions are dominant, ranging from 2 to 8%. In general, all predictions (based on the default $\alpha _\textrm{S} (m_{{\textrm{Z}}})$ for each PDF set) are in agreement with the measurement within the experimental and theoretical uncertainties.

## Determination of $\alpha _\textrm{S} (m_{{\textrm{Z}}})$

The sensitivity of the $R_{\varDelta \phi }(p_{\textrm{T}})$ ratio to the strong coupling constant is investigated by varying $\alpha _\textrm{S} (m_{{\textrm{Z}}})$ for each PDF set within the ranges presented in Table 3. The $\alpha _\textrm{S} (m_{{\textrm{Z}}})$ value in the fixed-order matrix elements calculations is also adjusted accordingly. Figure 9 shows the results for each PDF set, where the solid green (red) curves represent the maximum (minimum) $\alpha _\textrm{S} (m_{{\textrm{Z}}})$ values, and the dashed black curves correspond to intermediate $\alpha _\textrm{S} (m_{{\textrm{Z}}})$ values in $\varDelta \alpha _\textrm{S} (m_{{\textrm{Z}}}) = \pm 0.001$ or $\varDelta \alpha _\textrm{S} (m_{{\textrm{Z}}}) = \pm 0.002$ steps. A large sensitivity of $R_{\varDelta \phi }(p_{\textrm{T}})$ to variations of the strong coupling constant is observed for all PDF sets, and hence $R_{\varDelta \phi }(p_{\textrm{T}})$ can be used for the determination of $\alpha _\textrm{S} (m_{{\textrm{Z}}})$.

**Fig. 10:**
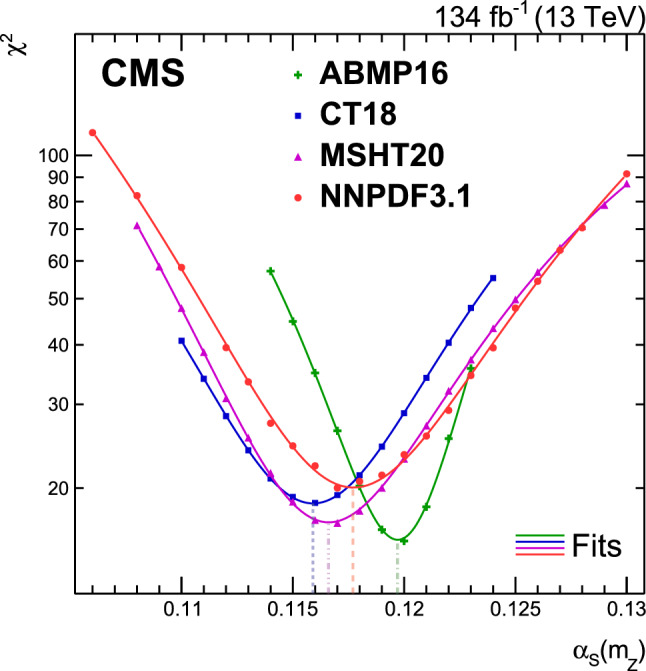
Minimization of the $\chi ^2$ between experimental measurements and theoretical predictions for the $R_{\varDelta \phi }(p_{\textrm{T}})$ ratio, with respect to $\alpha _\textrm{S} (m_{{\textrm{Z}}})$ for the ABMP16, CT18, MSHT20, and NNPDF3.1 NLO PDF sets. In this plot, only experimental uncertainties are included in the covariance matrix. The minimum value of $\alpha _\textrm{S} (m_{{\textrm{Z}}})$ found for each PDF set is indicated with a dashed line and corresponds to the central result. The experimental uncertainty is estimated from the $\alpha _\textrm{S} (m_{{\textrm{Z}}})$ values for which the $\chi ^2$ is increased by one unit with respect to the minimum value

**Table 4:** Results for $\alpha _\textrm{S} (m_{{\textrm{Z}}})$, associated uncertainties, and goodness-of-fit per degree of freedom ($\chi ^2/n_\text {dof}$), obtained from the measured $R_{\varDelta \phi }(p_{\textrm{T}})$ distribution compared with theoretical predictions using different NLO PDF sets

NLO PDF set	$\alpha _\textrm{S} (m_{{\textrm{Z}}})$	Exp.	NP	PDF	EW	Scale	$\chi ^2/n_\text {dof}$
ABMP16	0.1197	0.0008	0.0007	0.0007	0.0002	$_{-0.0042}^{+0.0043}$	16/16
CT18	0.1159	0.0013	0.0009	0.0014	0.0002	$_{-0.0067}^{+0.0099}$	19/16
MSHT20	0.1166	0.0013	0.0008	0.0010	0.0003	$_{-0.0063}^{+0.0112}$	17/16
NNPDF3.1	0.1177	0.0013	0.0011	0.0010	0.0003	$_{-0.0068}^{+0.0114}$	20/16

**Fig. 11:**
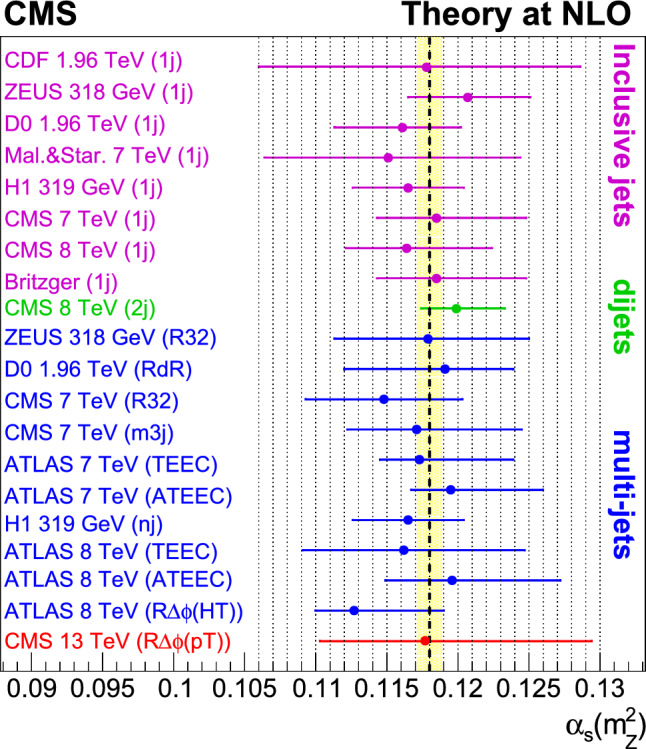
Determination of $\alpha _\textrm{S} (m_{{\textrm{Z}}})$ from the $R_{\varDelta \phi }(p_{\textrm{T}})$ ratio with the NNPDF3.1 PDF set (red), in comparison with previous NLO determinations of $\alpha _\textrm{S} (m_{{\textrm{Z}}})$ from inclusive jet (magenta), dijet (green), and multijet (blue) measurements. The horizontal error bars indicate the total uncertainty (experimental and theoretical). The world-average $\alpha _\textrm{S} (m_{{\textrm{Z}}})$ value is represented by the vertical dashed black line and its uncertainty by the yellow band

The value of $\alpha _\textrm{S} (m_{{\textrm{Z}}})$ is determined by minimising the goodness-of-fit ($\chi ^2$) between the experimental measurements and the theoretical predictions. The $\chi ^2$ is defined as:

9 $$\begin{aligned} \chi ^2 = \sum \limits _{ij}^N(D_{i}-T_{i})C_{ij}^{-1}(D_{j}-T_{j}), \end{aligned}$$

where *N* is the number of measurements, $D_{i}$ are the experimental measurements, $T_{i}$ are the theoretical predictions and $C_{ij}$ is the covariance matrix, which is composed of:

10 $$\begin{aligned} C= &   C_{\text {stat}}+C_{\text {uncor}}+\left( \sum \limits _{\text {sources}}C_{\text {JES}}\right) +C_{\text {unfold}}\nonumber \\  &   +C_{\text {pref}}+C_{\text {NP}}+C_{\text {PDF}}+C_{\text {EW}}, \end{aligned}$$

where $C_{\text {stat}}$ represents the statistical uncertainty, $C_{\text {uncor}}$ is the numerical precision of the fixed-order predictions, which is assigned as uncorrelated uncertainty to each bin, $C_{\text {JES}}$ is the systematic uncertainty for each JES uncertainty source, $C_{\text {unfold}} = C_{\text {JER}} + C_{\text {PU}} + C_{\text {MC}_\text {model}}$ is the systematic uncertainty induced through unfolding (representing the JER, pileup, and model uncertainties, respectively, described in Sect. 4.4), $C_{\text {pref}}$ is the trigger prefiring uncertainty [45], and $C_{\text {NP}}$, $C_{\text {PDF}}$, and $C_{\text {EW}}$ are the NP, PDF, and EW uncertainties, respectively.

The JES, unfolding, prefiring, NP, PDF, and EW uncertainties are considered as 100% correlated among $p_{\textrm{T}}$ bins, and are treated as multiplicative. Including only the experimental (statistical, JES, unfolding, and prefiring) uncertainties in the covariance matrix composition, the central $\alpha _\textrm{S} (m_{{\textrm{Z}}})$ result is obtained by minimising the $\chi ^2$ with respect to $\alpha _\textrm{S} (m_{{\textrm{Z}}})$. The associated experimental uncertainty is estimated from the $\alpha _\textrm{S} (m_{{\textrm{Z}}})$ values, for which the $\chi ^2$ is increased by one unit with respect to the minimum value. Figure 10 illustrates the $\chi ^2$ minimization curves for each PDF set, which result in the $\alpha _\textrm{S} (m_{{\textrm{Z}}})$ values and their respective experimental uncertainties listed in Table 4.

The propagation of NP, PDF, and EW uncertainties is estimated separately by repeating the $\chi ^2$ minimization procedure after including the relevant terms in Eq. (10). For the evaluation of scale uncertainties, the $\chi ^2$ comparison between measurement and theoretical predictions is repeated for the six different combinations of $\mu _\textrm{R}$ and $\mu _\textrm{F}$ scales described in Sect. 5. The up/down scale uncertainties correspond to the difference between the highest/lowest and the nominal $\alpha _\textrm{S} (m_{{\textrm{Z}}})$ values, respectively. All resulting $\alpha _\textrm{S} (m_{{\textrm{Z}}})$ values for the different PDF sets are fully compatible among each other, as well as with the world average [5]. The spread of these $\alpha _\textrm{S} (m_{{\textrm{Z}}})$ values from the different PDF sets shown in Table 4, is used for the assignment of an additional uncertainty in the final $\alpha _\textrm{S} (m_{{\textrm{Z}}})$ result due to the PDF choice. This uncertainty is evaluated from the maximum difference among the $\alpha _\textrm{S} (m_{{\textrm{Z}}})$ values determined using the NNPDF3.1 NLO PDF set, and all the other $\alpha _\textrm{S} (m_{{\textrm{Z}}})$ values determined using the other PDF sets shown in Table 4. The final result from the present analysis using the NNPDF3.1 NLO PDF set is: $\alpha _\textrm{S} (m_{{\textrm{Z}}}) = 0.1177_{-0.0068}^{+0.0114}~\,\text {(scale)} \pm 0.0013~\,\text {(exp)} \pm 0.0011~\,\text {(NP)} \pm 0.0010~\,\text {(PDF)} \pm 0.0003~\,\text {(EW)} \pm 0.0020~\,\text {(PDF choice)}$. This result, in comparison with a selection of $\alpha _\textrm{S} (m_{{\textrm{Z}}})$ determinations at NLO accuracy obtained from inclusive jet [7, 13, 15, 20, 24, 90–92], dijet [25], and multijet [7, 9, 16, 17, 19, 23, 91, 93–95] measurements is presented in Fig. 11.

**Table 5:** Values of $\alpha _\textrm{S} (m_{{\textrm{Z}}})$ and $\alpha _\textrm{S} (Q)$ determined in four different jet $p_{\textrm{T}}$ fitting subregions corresponding to an average scale $\langle Q \rangle$ over each $p_{\textrm{T}}$ interval

$p_{\textrm{T}}$ range ($\text {Ge}\hspace{-.08em}\text {V}$)	$\alpha _\textrm{S} (m_{{\textrm{Z}}})$	$\langle Q \rangle$ ($\text {Ge}\hspace{-.08em}\text {V}$)	$\alpha _\textrm{S} (Q)$
360–700	$0.1177_{-0.0067}^{+0.0104}$	433.0	$0.0967_{-0.0044}^{+0.0066}$
700–1190	$0.1162_{-0.0073}^{+0.0108}$	819.0	$0.0878_{-0.0042}^{+0.0060}$
1190–1870	$0.1159_{-0.0077}^{+0.0112}$	1346.0	$0.0830_{-0.0040}^{+0.0055}$
1870–3170	$0.1118_{-0.0070}^{+0.0110}$	2081.0	$0.0775_{-0.0034}^{+0.0051}$

**Fig. 12:**
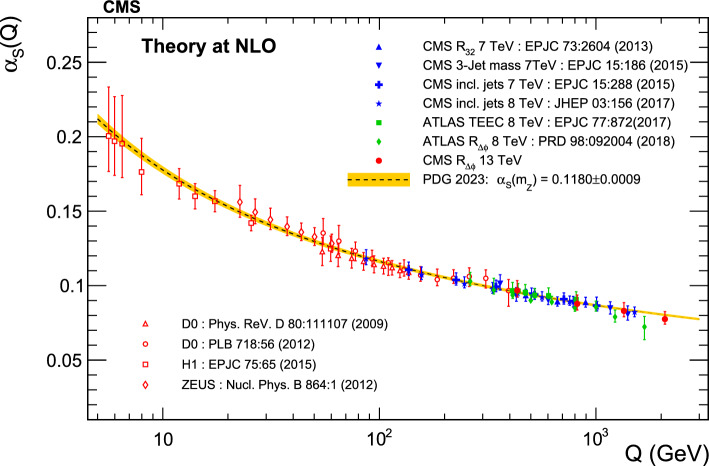
Running of the strong coupling $\alpha _\textrm{S} (Q)$ (dashed line) evolved using the current world-average value $\alpha _\textrm{S} (m_{{\textrm{Z}}}) = 0.1180 \pm 0.0009$ [5] together with its associated total uncertainty (yellow band). The four new extractions from the present analysis (Table 5) are shown as filled red circles, compared with results from the H1 [91, 94, 95], ZEUS [96], D0 [7, 14], CMS [16, 19, 20, 24], and ATLAS [9, 23] experiments. The vertical error bars indicate the total uncertainty (experimental and theoretical). All the experimental results shown in this figure are based on fixed-order predictions at NLO accuracy in pQCD

For the investigation of the running of the strong coupling, the fitted region of $p_{\textrm{T}} = 360$–3170$\,\text {Ge}\hspace{-.08em}\text {V}$ (16 points) is split into four $p_{\textrm{T}}$ subregions: 360–700, 700–1190, 1190–1870, and 1870–3170$\,\text {Ge}\hspace{-.08em}\text {V}$ (4 points each). The fitting procedure is repeated and the $\alpha _\textrm{S} (m_{{\textrm{Z}}})$ and all the relevant uncertainties are determined in each subregion separately. The $\alpha _\textrm{S} (m_{{\textrm{Z}}})$ values from each subregion are evolved to $\alpha _\textrm{S} (Q)$, where *Q* is chosen as the jet $p_{\textrm{T}}$ and is calculated as a cross-section-weighted average $(\langle Q \rangle )$ for each subregion. This study is performed using the NNPDF3.1 NLO PDF set. The values of $\alpha _\textrm{S} (m_{{\textrm{Z}}})$ and the results for $\alpha _\textrm{S} (Q)$ evaluated at the respective $\langle Q \rangle$ for each fitted subregion are shown in Table 5.

Figure 12 shows the energy dependence predicted by the RGE (dashed line) using the current world-average value $\alpha _\textrm{S} (m_{{\textrm{Z}}}) = 0.1180 \pm 0.0009$ [5] together with its associated total uncertainty (yellow band). The results from the $\alpha _\textrm{S} (Q)$ determinations in the four subregions presented in Table 5 are also shown, along with $\alpha _\textrm{S}$ values determined at lower scales by the H1 [91, 94, 95], ZEUS [96], D0 [7, 14], CMS [16, 19, 20, 24], and ATLAS [9, 23] Collaborations. All results reported in this study are consistent with the energy dependence predicted by the RGE, and no deviation is observed from the expected behaviour up to $\sim 2\,\text {Te}\hspace{-.08em}\text {V}$.

## Summary

A measurement of the $R_{\varDelta \phi }(p_{\textrm{T}})$ ratio, sensitive to azimuthal correlations in multijet events, has been presented using proton-proton collision data collected by the CMS experiment at a centre-of-mass energy of 13$\,\text {Te}\hspace{-.08em}\text {V}$ and corresponding to an integrated luminosity of 134$\,\text {fb}^{-1}$. The experimental data are compared with predictions from Monte Carlo (MC) event generators, pythia8 with tunes CUETP8M1 and CUETP8M2, herwig++ with tune UE-EE-5-CTEQ6L1, and powheg interfaced with each one of them. Deviations between data and MC predictions are observed in all cases, except for pythia8 tune CUETP8M2, which gives a good overall description of the measurement.

The measurement is also compared with fixed-order perturbative quantum chromodynamics (pQCD) predictions at next-to-leading-order (NLO) accuracy using the NLOJet++ package within the fastNLO framework. Those predictions are extracted for four different NLO parton distribution function (PDF) sets, ABMP16, CT18, MSHT20, and NNPDF3.1. Corrections for nonperturbative (NP) effects are evaluated using all the aforementioned MC event generators, and are applied to the fixed-order predictions. The predictions are additionally corrected for electroweak (EW) effects that become important at large jet transverse momenta. Generally, the fixed-order predictions are in agreement with the experimental data in the phase space of this analysis, and they provide a good description of the measured $R_{\varDelta \phi }(p_{\textrm{T}})$ distribution for all PDF sets.

Based on a comparison of the measured $R_{\varDelta \phi }(p_{\textrm{T}})$ distribution and the theoretical predictions, the strong coupling at the scale of the Z boson mass is: $\alpha _\textrm{S} (m_{{\textrm{Z}}}) = 0.1177_{-0.0068}^{+0.0114}\,\text {(scale)}\pm 0.0013\,\text {(exp)}\pm 0.0011\,\text {(NP)}\pm 0.0010\, \text {(PDF)}\pm 0.0003\,\text {(EW)}\pm 0.0020\,\text {(PDF choice)} = 0.1177_{-0.0074}^{+0.0117}$, using calculations based on the NNPDF3.1 NLO PDF set. Alternative $\alpha _\textrm{S} (m_{{\textrm{Z}}})$ results obtained with other PDF sets are compatible among each other, as well as with the central result of this work, and with the current world average, $\alpha _\textrm{S} (m_{{\textrm{Z}}}) = 0.1180\pm 0.0009$. The spread of the $\alpha _\textrm{S} (m_{{\textrm{Z}}})$ values obtained from different PDF sets is used for the assignment of the “PDF choice” uncertainty quoted in the final strong coupling constant derived here. The dominant uncertainty in this measurement originates from the scale dependence of the NLO pQCD predictions, and is expected to be significantly reduced with the future inclusion of fixed-order predictions at next-to-NLO accuracy.

The evolution of the strong coupling as a function of the energy scale, $\alpha _\textrm{S} (Q)$, has been tested up to $Q\approx 2\,\text {Te}\hspace{-.08em}\text {V}$, a higher scale than that probed in previous H1, ZEUS, D0, CMS, and ATLAS measurements. This test has been performed by choosing as energy scale *Q* the average jet transverse momentum in the different intervals considered, and no deviation from the expected NLO pQCD running of the strong coupling is observed.

## Data Availability

Data cannot be made available for reasons disclosed in the data availability statement. [Authors’ comment: Release and preservation of data used by the CMS Collaboration as the basis for publications https://cms-docdb.cern.ch/cgi-bin/PublicDocDB/RetrieveFile?docid=6032&filename=CMSDataPolicyV1.2.pdf &version=2 CMS data preservation, re-use and open access policy.]

## Code Availability Statement

This manuscript has associated code/software in a data repository. [Authors’comment: The CMS core software is publicly available on GitHub (https://github.com/cms-sw/cmssw).]
